# Unravelling the diversity of magnetotactic bacteria through analysis of open genomic databases

**DOI:** 10.1038/s41597-020-00593-0

**Published:** 2020-07-31

**Authors:** Maria Uzun, Lolita Alekseeva, Maria Krutkina, Veronika Koziaeva, Denis Grouzdev

**Affiliations:** 1 Research Center of Biotechnology of the Russian Academy of Sciences, Institute of Bioengineering, Moscow, Russia; 2 Lomonosov Moscow State University, Moscow, Russia

**Keywords:** Bacterial genetics, Microbial ecology, Evolutionary genetics, Data mining

## Abstract

Magnetotactic bacteria (MTB) are prokaryotes that possess genes for the synthesis of membrane-bounded crystals of magnetite or greigite, called magnetosomes. Despite over half a century of studying MTB, only about 60 genomes have been sequenced. Most belong to *Proteobacteria*, with a minority affiliated with the *Nitrospirae*, *Omnitrophica*, *Planctomycetes*, and *Latescibacteria*. Due to the scanty information available regarding MTB phylogenetic diversity, little is known about their ecology, evolution and about the magnetosome biomineralization process. This study presents a large-scale search of magnetosome biomineralization genes and reveals 38 new MTB genomes. Several of these genomes were detected in the phyla *Elusimicrobia*, *Candidatus* Hydrogenedentes, and *Nitrospinae*, where magnetotactic representatives have not previously been reported. Analysis of the obtained putative magnetosome biomineralization genes revealed a monophyletic origin capable of putative greigite magnetosome synthesis. The ecological distributions of the reconstructed MTB genomes were also analyzed and several patterns were identified. These data suggest that open databases are an excellent source for obtaining new information of interest.

## Introduction

The amount of data obtained from genome and metagenome sequencing has been sharply increasing for the last several years^1^. These data are kept in open databases, such as the widely used NCBI^2^ and IMG^3^ databases. In the case of IMG, the number of entries for metagenomic data greatly exceeds that for genomic ones^3^. In most cases, scientists use only a part of the sequencing information uploaded to the databases, leaving large quantities of information essentially unanalyzed. This gives the possibility that the obtained data may contribute to other studies and shorten the time and efforts of other scientists. In the present study, data stored in open genomic and metagenomic databases were used to search for magnetosome biomineralization genes related to magnetotactic bacteria (MTB).

The MTB are a group of organisms characterized by the ability to synthesize magnetosomes, which are crystals of magnetite (Fe_3_O_4_) or greigite (Fe_3_S_4_) enveloped by a lipid membrane^4^. These crystals can be applied in medicine as contrast agents for MRI^5^ and for treating tumors using magnetic hyperthermia^6^, and they are also of great interest in geology^7–9^ and astrobiology^10^. The synthesis of magnetosomes is controlled by the magnetosome gene cluster (MGC), previously called the magnetosome island or MAI. The MGC comprises genes that control magnetosome biosynthesis and that determine magnetosome morphology and chemical composition. The MGCs are unique and are associated only with MTB. The genes essential to the biomineralization process are called *mam* (magnetosome membrane) genes. Nine of them (*mamA, -В, -M, -K, -P, -Q, -E, -O*, and *-I*), are present in all MGCs^11,12^. In addition to the *mam* genes, genes specific to certain groups may also occur; for instance, *mad* genes are found in MTB from the *Deltaproteobacteria* and *Nitrospirae*, while *man* genes are present only in the *Nitrospirae*^13^.

At present, only about 60 MTB genomes are known, and most are affiliated with the phyla *Proteobacteria*, *Nitrospirae*, and *Ca*. Omnitrophica. Recently, MTB genomes associated with *Latescibacteria*^14^ and *Planctomycetes*^12^ have been found in open databases, implying that these databases could contain substantial amounts of new information about MTB.

To date, due to the lack of sufficient amounts of genomic data, little is known about the origin and evolution of MGCs^15^. Thus, additional investigations are needed to determine the mono- or polyphyletic origin of the MGCs, their evolutionary history, and whether the original MGCs were responsible for magnetite or greigite biomineralization.

This article describes the first large-scale search of magnetosome biomineralization genes in open genomic and metagenomic databases. Bioinformatics analysis of the search results allowed new MTB genomes to be obtained. Taxonomic assignments for the studied genomes provided the first evidence of their affiliation to new for MTB taxonomic ranks, including three new phyla. These results significantly expanded the knowledge of MTB diversity. The analysis of the ecological distribution of the reconstructed MTB genomes helped to identify several new patterns. Further comparative analysis of MGCs and marker genes of studied genomes allowed new data to be obtained concerning the origin and evolution of magnetosome biomineralization genes.

## Results

### The search for magnetosome biomineralization genes in open databases

The search for MTB genomes in open databases was guided by detecting MGCs unique to magnetotactic bacteria. Unfortunately, MGC sequences are not annotated as magnetosomal in open databases. This necessitated the use of previously known sequences of MGCs as search targets. The search was further complicated by the low identity values between the sequences of the same MGC gene in different MTB taxonomic groups. To cover the maximum number of new MTB representatives, MGC protein sequences were drawn from all known taxonomic groups where MTB were found previously. For this purpose, a database was created of known MGC protein sequences^12–43^ (Supplementary Table S1). The database included 67 MGCs from *Proteobacteria, Nitrospirae, Ca*. Omnitrophica, *Latescibacteria*, and *Planctomycetes*. The sequences of nine Mam proteins present in all MGCs were used to conduct BLASTp with genomic data from the NCBI and IMG databases. This resulted in the detection of four new genomes containing magnetosome biomineralization genes (Table 1, Supplementary Table S2).

**Table 1:** Characteristics of genomes with MGCs obtained from the NCBI and IMG database genomic data.

Organism	Phylum/Class	Accession in NCBI/IMG	Size (bp)	Scaffolds (no.)	GC (%)	N50 (bp)	CheckM completeness (%)	CheckM contamination (%)
Magnetovibrio sp. ARS8^51,83^	*Alphaproteobacteria*	GCA_002686765.1	2019305	197	59.64	10605	62.87	1.00
Elusimicrobia bacterium NORP122^64,84^	*Elusimicrobia*	GCA_002401485.1	2913226	191	54.93	19622	74.06	1.82
Unclassified Nitrospina Bin 25^45,114^	*Nitrospinae*	2651870060	4158979	431	37.69	11956	92.31	4.27
Planctomycetes bacterium SCGC_JGI090-P21^115^	*Planctomycetes*	2264265205	1230646	242	49.20	12722	38.87	2.19

**Fig. 1:**
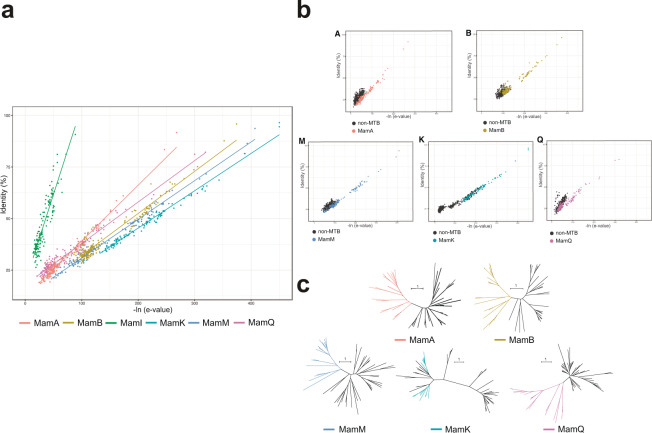
The choice of Mam protein for further searching for MGCs in open databases. (**a**) Correlations between –ln of e-values (x axis) and identities (y axis) among MamA, -B, -M, -K, -I, and -Q proteins sequences. (**b**) Correlations between identities and –ln of e-values among Mam protein sequences with their homologs. (**c**) Phylogenetic trees based on investigated sequences. Trees were reconstructed by the maximum-likelihood method with LG + F + I + G4 substitution model. Bootstrap values were calculated based on 1000 resamplings. Bar represents one substitution per 100 amino acid positions.

The use of all nine Mam proteins in metagenomic databases is complicated by the fact that much more data is kept in metagenomic than in genomic ones. To hasten the search process, one Mam protein out of nine common ones that met the required parameters was chosen for further BLAST analysis. The first chosen parameter was the identity between sequences from different taxonomic groups in each protein. The low values of these identities allowed exclusion of MamE, MamO, and MamP proteins from the analysis. The remaining MamA, -B, -M, -K, -I, and -Q proteins were assessed for sequences with the highest -ln of e-values, in addition to high identities (Fig. 1a). MamI was the least consistent with these requirements and was not used in further analyses. By contrast, MamK was the most consistent.

Each Mam protein has its homologs in non-MTB that are not involved in the magnetosomes biomineralization process. These homologs should be avoided when searching for MGCs. For this, Mam protein was chosen whose identities and -ln of e-values were significantly varied from these parameters in homologs (Fig. 1b). MamK showed the best result in this case, and its minimum identity and –ln e-value between sequences were 30 and 135, respectively. However, part of homologs had identities and –ln of e-values similar to the values found between Mam protein sequences. These homologs were confirmed not to be Mam sequences by verifying their phylogenetic separation (Fig. 1c). The sequences of each Mam protein formed monophyletic clades, while MamK formed two clades. Despite this, no homologs were observed inside the MamK clades. Based on all the investigated parameter results, the MamK protein sequences were chosen for the MGC gene search in the open databases.

The MamK protein sequences were used for BLAST for 10587 metagenomes from water, terrestrial, engineered, and host-associated ecosystems. The analysis revealed 2798 sequences potentially affiliated with the MamK protein (Supplementary Fig. S1a). Their scaffolds were checked for the presence of other Mam protein sequences. After that, 227 MamK sequences referring to 135 metagenomes were obtained (Supplementary Tables S3 and S4). These and previously known MamK sequences were used to construct a phylogenetic tree (Supplementary Fig. S1b), which revealed that the identified MamK sequences were not closely related to previously known sequences. This assumes that they could refer to taxonomic groups in which MTB were not found before.

### Metagenome binning, phylogenomic inferences, and MGC reconstruction

The phylogenetic position of genomes to which the MamK sequences belonged was assessed by conducting metagenome binning, and it yielded 14688 metagenome-assembled genomes (MAGs) (Supplementary Table S3). Two metagenomes were also determined to be single-cell amplified genomes (SAGs), so no binning procedures were required for them. Of all the MAGs obtained in this study, only 140 contained previously detected MamK sequences. For those of the 140 whose completeness was >45% decontamination was conducted. This left 32 MAGs with completeness >45% and contamination <10% that contained MGCs (Table 2, Supplementary Table S6). The phylogenomic affiliations of the obtained MAGs, SAGs, and genomes were then determined, the MGСs genes were reconstructed, and the ecological distributions were studied.

**Table 2:** Characteristics of reconstructed MAGs with MGCs obtained from the IMG metagenomic data.

Organism	Phylum/Class	Metagenome accession in NCBI/IMG	Size (bp)	Scaffolds (no.)	GC (%)	N50 (bp)	CheckM completeness (%)	CheckM contamination (%)
Ca. Hydrogenedentes bacterium MAG_17963_hgd_111^85^	*Ca*. Hydrogenedentes	3300017963	3018788	288	60.18	11662	71.11	1.46
Ca. Hydrogenedentes bacterium MAG_17971_hgd_130^44^	*Ca*. Hydrogenedentes	3300017971	2683901	240	60.43	12541	60.01	1.16
Deltaproteobacteria bacterium MAG_00134_naph_006^86,119^	*Deltaproteobacteria*	3300000134	1498667	692	49.54	2676	60.69	3.87
Deltaproteobacteria bacterium MAG_00241_naph_010^87,119^	*Deltaproteobacteria*	3300000241	1547003	324	49.45	6761	55.59	2.41
Deltaproteobacteria bacterium MAG_00792_naph_016^88,119^	*Deltaproteobacteria*	3300000792	3032840	409	49.74	11269	89.28	5.86
Deltaproteobacteria bacterium MAG_09788_naph_37^89^	*Deltaproteobacteria*	3300009788	899797	137	47.24	7579	49.08	0.97
Deltaproteobacteria bacterium MAG_15370_dsfb_81^90,120^	*Deltaproteobacteria*	3300015370	3868622	334	48.42	14397	89.68	5.59
Deltaproteobacteria bacterium MAG_17929_sntb_26^91^	*Deltaproteobacteria*	3300017929	2777907	276	53.10	17193	62.13	5.10
Deltaproteobacteria bacterium MAG_17996_sntb_20^92^	*Deltaproteobacteria*	3300017996	1691080	454	53.11	4033	50.53	2.33
Deltaproteobacteria bacterium MAG_22204_dsfv_001^93^	*Deltaproteobacteria*	3300022204	2675335	75	52.74	60141	89.52	0.36
Deltaproteobacteria bacterium MAG_22309_dsfv_022^48^	*Deltaproteobacteria*	3300022309	2902378	66	55.15	78905	91.60	1.79
Gammaproteobacteria bacterium MAG_00150_gam_010^94^	*Gammaproteobacteria*	3300000150	2847655	486	49.07	8986	98.17	3.96
Gammaproteobacteria bacterium MAG_00160_gam_009^95^	*Gammaproteobacteria*	3300000160	2903803	318	49.10	15339	99.39	4.88
Gammaproteobacteria bacterium MAG_00172_gam_018^96^	*Gammaproteobacteria*	3300000172	2866084	274	48.97	18904	96.95	3.05
Gammaproteobacteria bacterium MAG_00188_gam_006^97^	*Gammaproteobacteria*	3300000188	2672010	567	48.83	6818	95.12	4.19
Gammaproteobacteria bacterium MAG_00212_gam_1^98^	*Gammaproteobacteria*	3300000212	2103212	955	48.40	2901	78.43	5.08
Gammaproteobacteria bacterium MAG_00215_gam_020^99^	*Gammaproteobacteria*	3300000215	2931288	507	49.02	8845	95.73	5.34
Magnetococcales bacterium MAG_21055_mgc_1^100^	*Ca*. Etaproteobacteria	3300021055	3585593	930	52.41	5203	84.82	3.65
Nitrospinae bacterium MAG_09705_ntspn_70^101^	*Nitrospinae*	3300009705	2024644	120	42.63	30902	67.25	2.56
Nitrospirae bacterium MAG_10313_ntr_31^102^	*Nitrospirae*	3300010313	1933163	344	35.33	7568	90.20	3.64
Pelobacteraceae bacterium MAG_21601_9_030^103^	*Deltaproteobacteria*	3300021601	2536371	232	54.11	20074	78.15	8.39
Pelobacteraceae bacterium MAG_13126_9_058^104^	*Deltaproteobacteria*	3300013126	3576562	72	52.01	83631	91.61	1.29
Pelobacteraceae bacterium MAG_21600_9_004^105^	*Deltaproteobacteria*	3300021600	3430740	60	51.50	87025	90.32	0.65
Planctomycetes bacterium MAG_11118_pl_115^106^	*Planctomycetes*	3300011118	3767441	157	48.98	33372	89.44	1.24
Planctomycetes bacterium MAG_17991_pl_60^107^	*Planctomycetes*	3300017991	1289005	144	49.53	10179	64.20	0.00
Planctomycetes bacterium MAG_18080_pl_157^108^	*Planctomycetes*	3300018080	3144921	139	48.44	34208	90.91	3.41
Rhodospirillaceae bacterium MAG_01419_mvb_30	*Alphaproteobacteria*	3300001419	2811682	477	55.72	7268	94.58	4.10
Rhodospirillaceae bacterium MAG_04806_tlms_2^109^	*Alphaproteobacteria*	3300004806	2085124	309	57.51	8435	87.64	2.12
Rhodospirillaceae bacterium MAG_05422_2-02_14^110^	*Alphaproteobacteria*	3300005422	2281835	255	61.09	11800	85.45	0.50
Rhodospirillaceae bacterium MAG_05596_2-02_51^111^	*Alphaproteobacteria*	3300005596	1831947	329	61.19	6777	76.91	0.25
Rhodospirillaceae bacterium MAG_06104_tlms_034^112^	*Alphaproteobacteria*	3300006104	3186839	353	64.25	13005	89.59	2.53
Rhodospirillaceae bacterium MAG_22225_2-02_112^113^	*Alphaproteobacteria*	3300022225	2547095	147	61.01	26510	91.17	5.22
*Ca*. Omnitrophica bacterium SCGC AG-290-C17 (SAG)^116^	*Ca*. Omnitrophica	3300015153	1712617	171	48.60	13921	62.84	0.00
Uncultured microorganism SbSrfc.SA12.01.D19 (SAG)^117^	*Deltaproteobacteria*	3300022116	2501480	175	52.60	25257	49.13	0.00

**Fig. 2:**
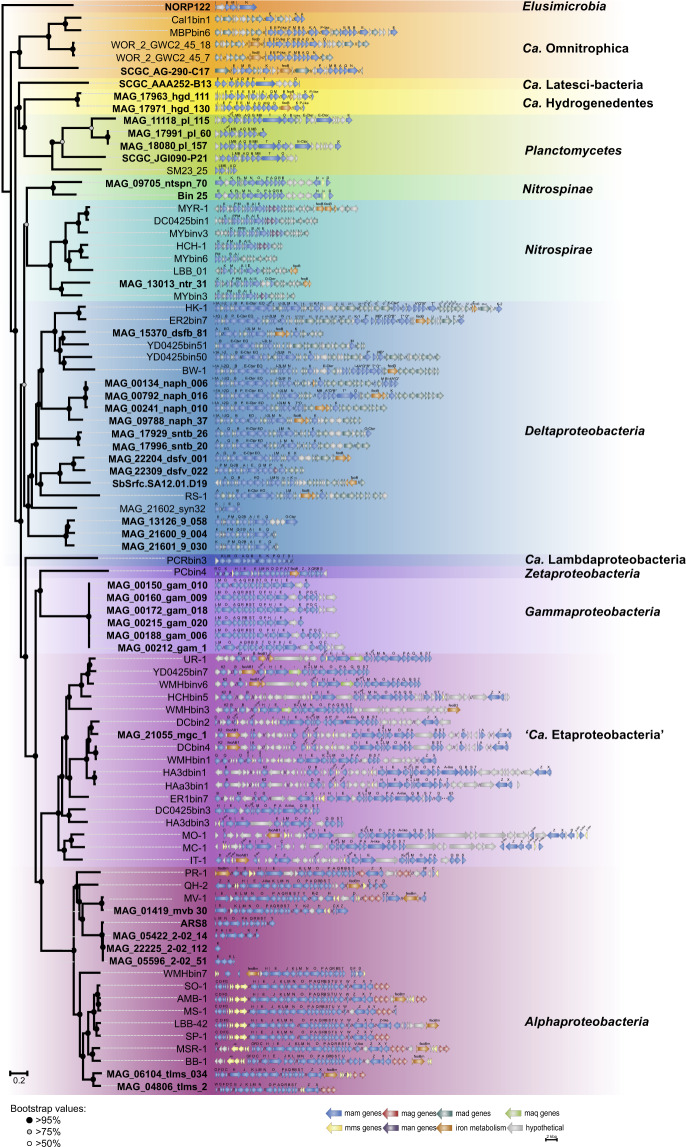
Comparison of the MGC regions in the MAGs and SAGs (in bold) obtained in this study versus previously known MTB genomes. Full names for MTB strains can be found in Supplementary Table S1.

The identification of the phylogenomic position of the studied genomes revealed, for the first time, their affiliation to the phyla *Elusimicrobia, Ca*. Hydrogenedentes, and *Nitrospinae* (Supplementary Fig. S2, Supplementary Tables S2 and S5). One genome was affiliated with the phylum *Elusimicrobia* and referred to order UBA1565 in the *Elusimicrobia* class. After MGC reconstruction, the *mamI, -B, -M*, and *-N* genes were revealed in the investigated genome (Fig. 2). Two MAGs from *Ca*. Hydrogenedentes belonged to the same species (98.70% average nucleotide identity), but they were obtained independently from different metagenomes. These MAGs referred to the GCA-2746185 family in the order *Hydrogenedentiales*. The 16S rRNA gene from the *Ca*. Hydrogenedentes bacterium MAG_17971_hgd_130^44^ had 90% similarity with the closest non-MTB *Ca*. Hydrogenedentes bacterium YC-ZSS-LKJ63. All these data confirmed that the obtained binning results were regular and did not represent a computational error. Only *mam* genes were found in the MGCs of the studied genomes.

In the *Nitrospinae* phylum, two MAGs were affiliated with different genera of the order *Nitrospinales*. Their MGCs revealed the presence of *mam* and *mms* (magnetic particle-membrane specific) genes. Samples for the metagenomes of the obtained MAGs were collected from the Gulf of Mexico^45^ and Arctic Ocean waters. Non-MTB representatives of this phylum were also detected only in marine habitats^46,47^, indicating that bacteria from the *Nitrospinae* could prefer to inhabit marine environments.

The 14 reconstructed MAGs belonged to different families of *Deltaproteobacteria*. Of the 14, three MAGs were affiliated with the UBA8499 genus in the *Pelobacteraceae* family. In their MGCs, apart from the *mam* and *mad* genes, which are typical for *Deltaproteobacteria*, the *man* genes were detected for the first time. Previously, the *man* genes were associated only with MTB from the *Nitrospirae*. Another two MAGs were affiliated with the *Syntrophobacteraceae* family, where MTB were discovered previously^41^. This is further evidence that binning was conducted correctly and that MTB representatives are indeed present in this family.

Three genomes also belonged to the *Desulfobulbales* order. Of these, the *Deltaproteobacteria* bacterium MAG_22309_dsfv_022^48^ contained *man3* gene in addition to the *mam* and *mad* genes, thereby confirming the routine presence of *man* genes in *Deltaproteobacteria*. A further four MAGs were related to the NaphS2 family in the *Desulfatiglanales* order. Analysis of their MGCs revealed genes responsible for putative greigite magnetosome synthesis. Metagenomic samples of the studied genomes were obtained from marine sediments, as well as all other known non-MTB genomes of this family^49,50^.

In *Alphaproteobacteria*, three MAGs and one genome were related to a 2-02-FULL-58-16 family in the *Rhodospirillales* order. Metagenomic samples of the studied genomes were isolated from marine ecosystems. The other non-MTB genomes of this family were also detected only in marine ecosystems^51^. For the first time, two MAGs containing MGCs were also detected in *Telmatospirillum* genus. Their metagenomic samples were collected from a freshwater bog. *Telmatospirillum siberiense*, the only known representative of this genus, was also isolated from freshwater peat soil^52^. Thus, this group possibly tends to inhabit freshwater ecosystems. Reconstruction of the MGCs revealed *mam* and *mms* genes in the studied MAGs. One MAG was referred to the *Ca*. Etaproteobacteria class. Genomes from this class previously were found in both saline and freshwater habitats^15,31,53^. The obtained MAG clustered with genomes isolated from freshwater environments. The MGC of the recovered MAG revealed a standard gene set inherent to MTB from this class. A further six MAGs were affiliated with the *Gammaproteobacteria*. All of these were sampled from one source and had 100% identity between their genes. Only the *mam* genes were detected in their MGCs.

The *Nitrospirae* phylum was affiliated with one MAG. A metagenomic sample of this phylum was obtained from a hot spring. Previously, other MTB and non-MTB from this phylum were also detected in hot springs^54,55^. Three of the recovered MAGs belonged to the SG8-4 order in the *Phycisphaerae* class of *Planctomycetes*. Apart from the reconstructed MAGs, one SAG was also obtained from the UBA1845 order in *Phycisphaerae* class. The completeness of this SAG was very low (39%), but it was also taken into analyses due to the large number of *mam* genes detected in the MGC. Another detected SAG was affiliated with *Сa*. Omnitrophica and was referred to the GWA2-52-8 family in the *Omnitrophales* order. The MGC of this genome had a set of genes that were specific to all magnetotactic representatives from this phylum.

### Reconstruction of the evolutionary pathways for MGCs

**Fig. 3:**
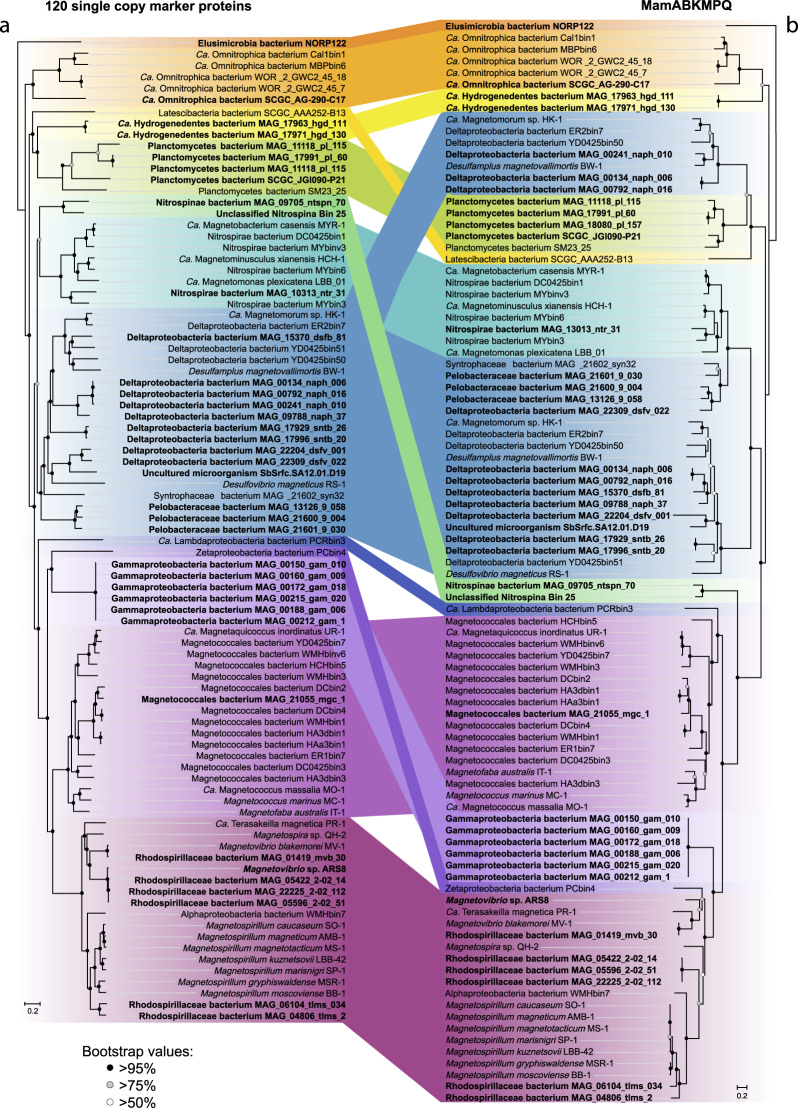
Maximum-likelihood phylogenomic trees of MTB genomes. Trees were inferred from a comparison of 120 concatenated single-copy marker proteins of MTB genomes (**a**) and concatenated magnetosome associated protein sequences (MamABKMPQ) (**b**). Both trees were reconstructed with evolutionary model LG + F + I + G4. Branch supports were obtained with 1000 ultrafast bootstraps. The scale bar represents amino acid substitutions per site.

The identification of putative genes involved in magnetosome biomineralization allowed investigation of MGC evolutionary pathways. These were analyzed by constructing a phylogenetic tree of concatenated MamABKMPQ sequences (“Mam tree”, Fig. 3b) and comparing this tree with one based on 120 single-copy marker gene proteins (“core genome tree”, Fig. 3a). Comparative analysis of the MTB position on the trees revealed some incongruences. For instance, the *Deltaproteobacteria* group from “core genome tree” was divided into three subgroups on the “Mam tree.” The first subgroup comprised representatives capable of putative greigite magnetosome synthesis, while the other two subgroups included representatives with MGCs for magnetite magnetosome biomineralization. One of the magnetite subgroups included representatives of the *Pelobacteraceae*, *Syntrophia*, and *Desulfurivibrionaceae* families, which clustered with the *Nitrospirae*. According to the “Mam tree” topology, the *man* genes could be assumed to have originated in the *Deltaproteobacteria* and were inherited by the *Nitrospirae* through horizontal gene transfer. The compared trees also indicated vertical inheritance in the *Alpha-* and *Ca*. Etaproteobacteria groups, although the occurrence of horizontal transfer events was previously established in these groups^27,31^. These types of transfers have been confirmed to have occurred recently, which is why they cannot be detected through the tree topology analysis.

A further investigation examined whether MGC originated once or more than once. This was done by adding the Mam protein sequences recovered in this study to previously known Mam protein sequences and their non-MTB homologs and then constructing phylogenetic trees (Supplementary Fig. S3). Analysis of the constructed trees confirmed the previous results^15^ showing that all Mam protein sequences, except for MamK, formed monophyletic clades and that these clades did not contain any homolog sequences. This indicates that the MGCs for magnetite and greigite synthesis are likely to have a common origin.

**Fig. 4:**
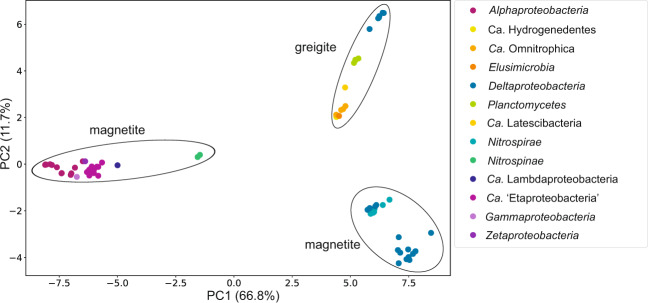
The prediction of magnetosome chemical composition for phyla in which MTB genomes were found for the first time. Predictions were made using principal component analysis for a maximum-likelihood distance matrix of concatenated Mam protein sequences.

The magnetosome chemical composition in genomes of every phylum where MTB were found for the first time were predicted by counting the phylogenetic distances of the concatenated sequences of six essential Mam proteins (MamA, -B, -K, -M, -P, and -Q) and conducting a principal component analysis (Fig. 4). All values clustered to three groups. First was the group that comprised *Planctomycetes*, and *Latescibacteria*, which are known to have genes for putative greigite magnetosome synthesis^12,14,56^. The NaphS2 family of *Deltaproteobateria, Ca*. Hydrogenedentes, *Сa*. Omnitrophica, and *Elusimicrobia* also clustered with this group. The other two groups comprised representatives with magnetite magnetosome synthesis genes. The first magnetite group included *Nitrospinae* and all classes of *Proteobacteria* where MTB were known. The exception was the remaining studied classes of *Deltaproteobateria*, which clustered with the second magnetite group, together with *Nitrospirae*.

## Discussion

This study represents the first large-scale search of magnetosome biomineralization genes in open databases. Bioinformatic analysis of the gathered data almost doubled the number of MTB genomes from the 60 previously known; 4 genomes, 2 SAGs, and 32 MAGs were obtained as a result of this research. Besides, analysis of the database of collected MGC protein sequences revealed MamK as the most appropriate protein for MGC searching in open databases. This finding will allow the use of these putative protein sequences as markers for MTB detection in environmental samples.

This study also provides the first description of magnetosome biomineralization genes in the genomes of *Elusimicrobia, Nitrospinae*, and *Ca*. Hydrogenedentes. Non-MTB representatives of *Elusimicrobia* phylum were previously found as free-living^57^ and ecto- and endosymbionts^58,59^ of multicellular eukaryotes. MTB living symbiotically with eukaryotes have been detected previously^60,61^. Further investigations are needed to solve the enigma of whether MTB from *Elusimicrobia* free-living or symbiotic organisms are.

To date, little is known about *Ca*. Hydrogenedentes, except for its genome presence^62–64^. More is known about *Nitrospinae*, where one axenic culture was previously described^65^. However, these reports do not give an extensive understanding of the capabilities of this phylum’s representatives. Thus, the detection of MGCs in genomes that belong to these phyla significantly supplements the knowledge of MTB diversity and evolution, while also providing new information about these phyla.

This work also gives much new information about groups where MTB were previously recognized. For instance, the relatively few genomes were affiliated with *Alpha-* and *Ca*. Etaproteobacteria, while the current belief is that representatives of these classes dominate among MTB in all natural environments^12^. In addition, within the *Alphaproteobacteria* class, the presence of MGCs was discovered for the first time in genomes belonging to the *Telmatospirillum* genus. This may indicate a common origin for magnetosome biomineralization genes among the *Magnetospirillum, Magnetospira*, and *Magnetovibrio* genera.

Furthermore, for the first time the presence of *man* genes was revealed in MGCs of the *Deltaproteobacteria*. Previously, these genes were found only in *Nitrospinae*. Whether horizontal gene transfer events occurred between representatives of these phylogenetic groups or their MGCs shared a common origin is not known. Further studies are required to determine which possibility is correct.

The genomes with magnetosome biomineralization genes obtained in this study allowed the investigation of the origin and evolution of the MGCs. A comparison of the “core genome” and “Mam” trees revealed clustering of the *Deltaproteobacteria* greigite subgroup sequences with the *Planctomycetes, Latescibacteria, Ca*. Hydrogenedentes, *Сa*. Omnitrophica, and *Elusimicrobia* phyla. Of these, *Latescibacteria*^14^ and *Planctomycetes*^12^ were already known to have MGCs for putative greigite synthesis. Note that *Ca*. Omnitrophica was also associated with the greigite subgroup, although it is believed that they biomineralize magnetite magnetosomes^43^. Such assumptions are based on *Сa*. Omnitrophus magneticus SKK-01 however, this genome is highly contaminated (Supplementary Table S1). Thus, further investigations are needed to study *Ca*. Omnitrophica magnetosome chemical composition.

In addition to all mentioned findings, the latest version of the bacterial tree of life^66^, based on GTDB R04-RS89 reference data (Supplementary Fig. S4) helped to reveal the most ancient phylum in which MTB representatives were known. It was indicated that the *Elusimicrobia* phylum is the most closely related to the last universal common ancestor (LUCA). If the MTB of this phylum are assumed capable of greigite magnetosome synthesis, then greigite MGCs could have appeared much earlier than commonly believed, and the first MTB could have greigite, not magnetite, MGCs. The other phyla with MTB representatives in the vicinity of LUCA are *Ca*. Omnitrophica and *Proteobacteria*, although *Nitrospirae* MTB was previously thought to be the most ancient^40^.

Considering the existing data regarding the presence of horizontal transfer events among MTB and analyzing the discrepancies in “core genome” and “Mam” trees, the proposal could be made that horizontal gene transfers occur much more often than previously thought and are of great importance in MGC evolution.

The genomes obtained in this work require further confirmation by morphological identification. Once confirmed, these data will allow a more thorough study of the contribution of vertical and horizontal gene transfer events with respect to MGC inheritance. The data obtained in the present work will allow the study of the environmental and metabolic preferences of newly discovered MTB genomes, which may become the key to isolating them in axenic cultures. Moreover, a detailed MGC analysis could help to find as yet unidentified genes that are involved in magnetosome synthesis and to reveal much about the biomineralization process.

Generally, in this work, it was shown that MamK is the most appropriate protein for MGCs detecting in open databases. The search results allowed to receive 38 new genomes containing MGCs, that were affiliated to both taxonomic groups where MTB were found before and three new phyla. Thus, received MTB genomes permitted to unravel the MTB diversity and can be used in further MTB studies or in receiving new information about these phyla. Also, a comparison of MTB position on “mam tree” and “core genome tree” helped to reveal signs of putative horizontal gene transfers. This led to assumptions that such MGC transfers could occur with higher frequency and probably play a much more important role in MGC evolution than it was previously thought. Moreover, a proposal was made that the origin of MGC probably is more ancient than it was suggested earlier and possibly was capable of greigite magnetosomes biomineralization rather than magnetite.

Thus, all received data allowed the expansion of knowledge about MTB diversity, ecology, and evolution and has opened up new opportunities for further searches for and investigations of magnetotactic bacteria.

## Materials and methods

### The search for magnetosome biomineralization genes in open databases

The search for magnetosome biomineralization genes was conducted by collecting a database of MGC protein sequences based on currently known MTB genomes (Supplementary Table S1). The search was provided using BLASTp analysis, with identity >30% and e-value >1e^−05^. Searches of the IMG and NCBI genomic databases used sequences of nine essential Mam proteins from different taxonomic groups as targets. The IMG metagenomic database was searched by BLASTp using MamK sequences. The sequences obtained from BLAST analysis were further checked to separate MGC proteins from their homologs. For this, each Mam protein sequence was checked for joint clustering on the phylogenetic trees. The presence of other Mam proteins in the same scaffold provided additional support for choosing those scaffolds for further analysis. The search was conducted in April 2018.

### Genome reconstruction and analyses

Metagenome assembled genome (MAG) reconstruction was conducted using the Busybee web^67^, Maxbin2^68^, and MyCC^69^ with standard parameters. The DAS Tool^70^ was used for choosing consensus assemblies for the obtained MAGs. Completeness and contamination values of genomes were obtained using lineage-specific marker genes and default parameters in CheckM v. 1.0.12^71^. RefineM v. 0.0.24^50^ was used to remove contamination based on taxonomic assignments. This process, called ‘decontamination’, involves the classification of obtained genes and scaffolds in each MAG relative to the gene base with a known taxonomic classification. After that, scaffolds with incongruent taxonomic classifications are removed from the MAGs. The quality metrics were assessed using the QUAST^72^ tool. The average nucleotide identity (ANI) was calculated using fastANI^73^. The MGCs were determined using local BLAST and comparison with reference sequences of magnetotactic bacteria.

### Phylogenetic analyses

Taxonomic assignments for the studied genomes 16S rRNA genes were obtained using the GTDB 16S r89 dataset in IDTAXA^74^. The GTDB-Tk v.0.1.3^75^ ‘classify_wf’ command was used to find 120 single-copy bacterial marker protein sequences, to construct their multiple alignments and to get the taxonomic assignment using the GTDB r86 database^76^. Amino acid sequence sets of the MamA, -B, -M, -K, -P, and -Q proteins were independently aligned using MAFFT^77^, curated with Gblocks v. 0.91b^78^ with an option that allows gap positions within the final blocks, and then concatenated. These Mam protein sequences were also used to build trees with their homologs. Maximum-likelihood trees were inferred with IQ-TREE^79^ using evolutionary models selected by ModelFinder^80^. Branch supports were obtained with 1000 ultrafast bootstraps^81^. Trees were visualized with iTOL v4^82^. The genomes of *Ca*. Omnitrophus magneticus SKK-01, *Ca*. Magnetoglobus multicellularis str. Araruama, *Ca*. Magnetobacterium bavaricum TM-1, and *Ca*. Magnetoovum chiemensis CS-04 were not subjected to phylogenetic analyses because they had failed the quality check (Supplementary Table S1). Taxonomic classification of the obtained genomes on phylum rank was performed using NCBI taxonomy; other ranks were named using GTDB.

## Supplementary information

Supplementary Information

## Data Availability

The genomes and metagenomes used during the current study are publicly available in NCBI (https://www.ncbi.nlm.nih.gov/)^44,48,83–113^ and IMG (https://img.jgi.doe.gov/cgi-bin/m/main.cgi)^114–117^ databases. Scaffolds of obtained MAGs could be found in Supplementary Table S6, hosted at figshare^118^. All data generated and analyzed in this study are also available in figshare^118^ and in supplementary information accompany this paper. Assembly of Rhodospirillaceae bacterium MAG_01419_mvb_30 could be found in RAST (https://rast.nmpdr.org/) using ‘guest’ as login and as password.

## Code availability

The following tools were used for the presented analysis and described in the main text:

Busybee web, Maxbin2, MyCC, and DAS Tool with standard parameters were used for the reconstruction of metagenome-assembled genomes (MAGs).

  1. Busybee web https://ccb-microbe.cs.uni-saarland.de/busybee

  2. Maxbin2 https://sourceforge.net/projects/maxbin2/

  3. MyCC https://sourceforge.net/projects/sb2nhri/files/MyCC/

  4. DAS Tool https://github.com/cmks/DAS_Tool

  5. CheckM was used to estimate obtained genomes completeness and contamination https://github.com/Ecogenomics/CheckM

  6. RefineM was used to remove contamination https://github.com/dparks1134/RefineM

  7. QUAST helped to access quality metrics http://cab.spbu.ru/software/quast/

  8. fastANI was used to calculate ANI https://github.com/ParBLiSS/FastANI

  9. IDTAXA helped to obtain taxonomic assignments for the studied genomes 16S rRNA genes http://www2.decipher.codes/Classification.html

10. GTDB-Tk was used to find 120 single-copy bacterial marker protein sequences, to construct their multiple alignments and to get the taxonomic assignment using the GTDB r86 database https://github.com/Ecogenomics/GTDBTk

11. MAFFT was used for aligning amino acid sequence sets of the MamA, -B, -M, -K, -P, and -Q proteins https://mafft.cbrc.jp/alignment/server/

12. Gblocks helped to curate sequences aligned in MAFFT http://molevol.cmima.csic.es/castresana/Gblocks_server.html

13. Phylogenetic trees were inferred with IQ-TREE http://www.iqtree.org/

14. Obtained trees were visualized with iTOL https://itol.embl.de/
