# Supplementary material for: Unravelling the diversity of magnetotactic bacteria through analysis of open genomic databases
Source: Sci Data. 2020 Jul 31;7:252. doi: 10.1038/s41597-020-00593-0 (PMC7449369; doi:10.1038/s41597-020-00593-0)
Supplement: Supplementary file 1 — Supplementary Information [file 41597_2020_593_MOESM1_ESM.pdf]

Supplementary information

UNRAVELLING THE DIVERSITY OF MAGNETOTACTIC BACTERIA THROUGH ANALYSIS OF OPEN GENOMIC DATABASES

Maria Uzun, Lolita Alekseeva, Maria Krutkina, Veronika Koziaeva and Denis Grouzdev

Content

Supplementary Figure S1. 1

Supplementary Figure S2. 3

Supplementary Figure S3. 4

Supplementary Figure S4. 5

Supplementary Table S1 6

Supplementary Table S2 12

Supplementary Table S3 14

Supplementary Table S4 20

Supplementary Table S5 29

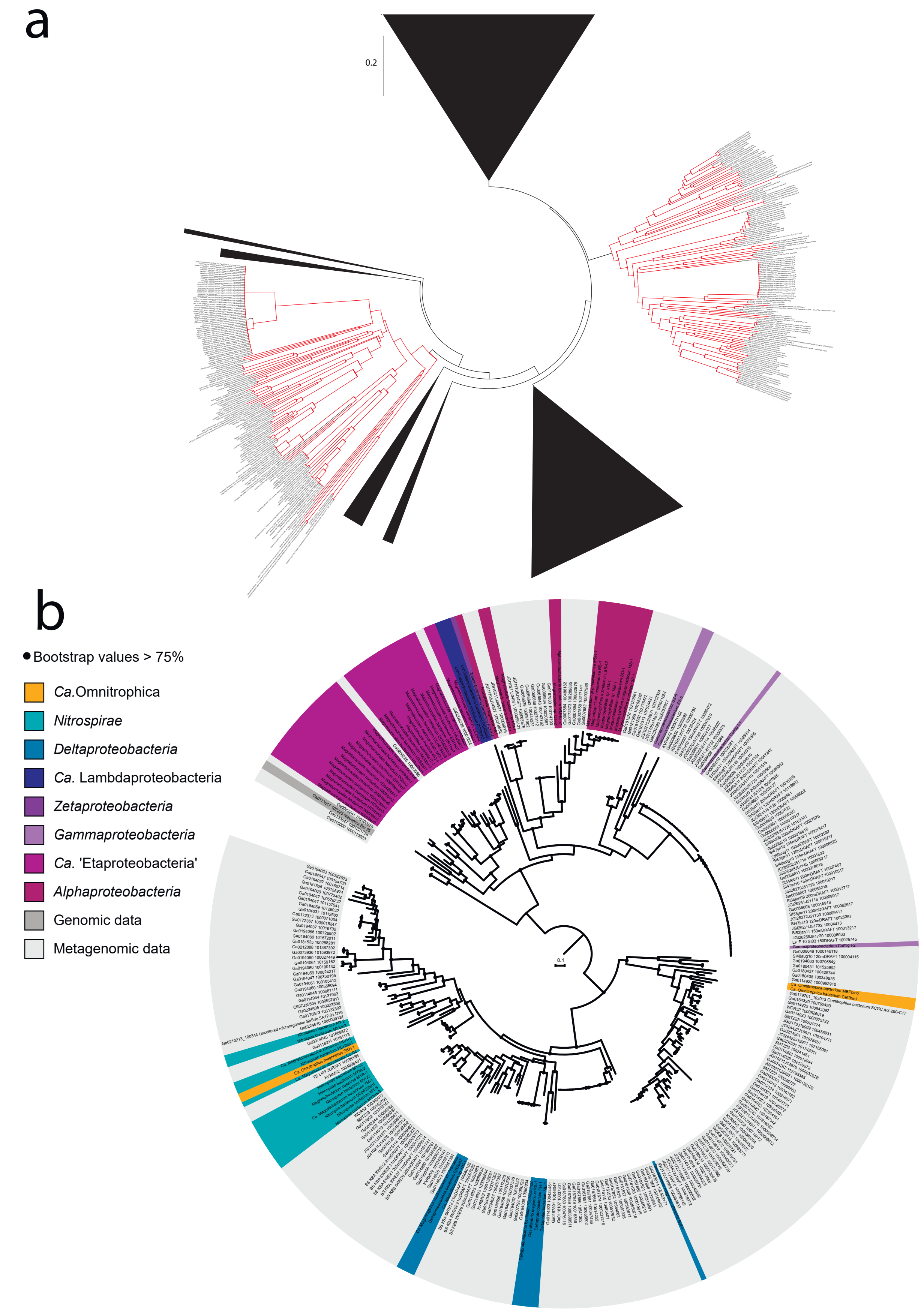

Supplementary Figure S1. Sequences obtained from the search for MamK protein in IMG database.
(a) Maximum-likelihood phylogenetic tree of 2798 sequences obtained from IMG database potentially affiliated with the MamK protein. Red-colored branches represent sequences, in the scaffolds of which the presence of other Mam proteins was detected. (b) Maximum-likelihood phylogenetic tree based on MamK sequences remained after checking for presence of other Mam proteins in scaffolds with MamK. Both trees were reconstructed with the evolutionary model LG+I+G4. Branch supports were obtained with 1000 ultrafast bootstraps. The scale bars represent amino acid substitutions per site.

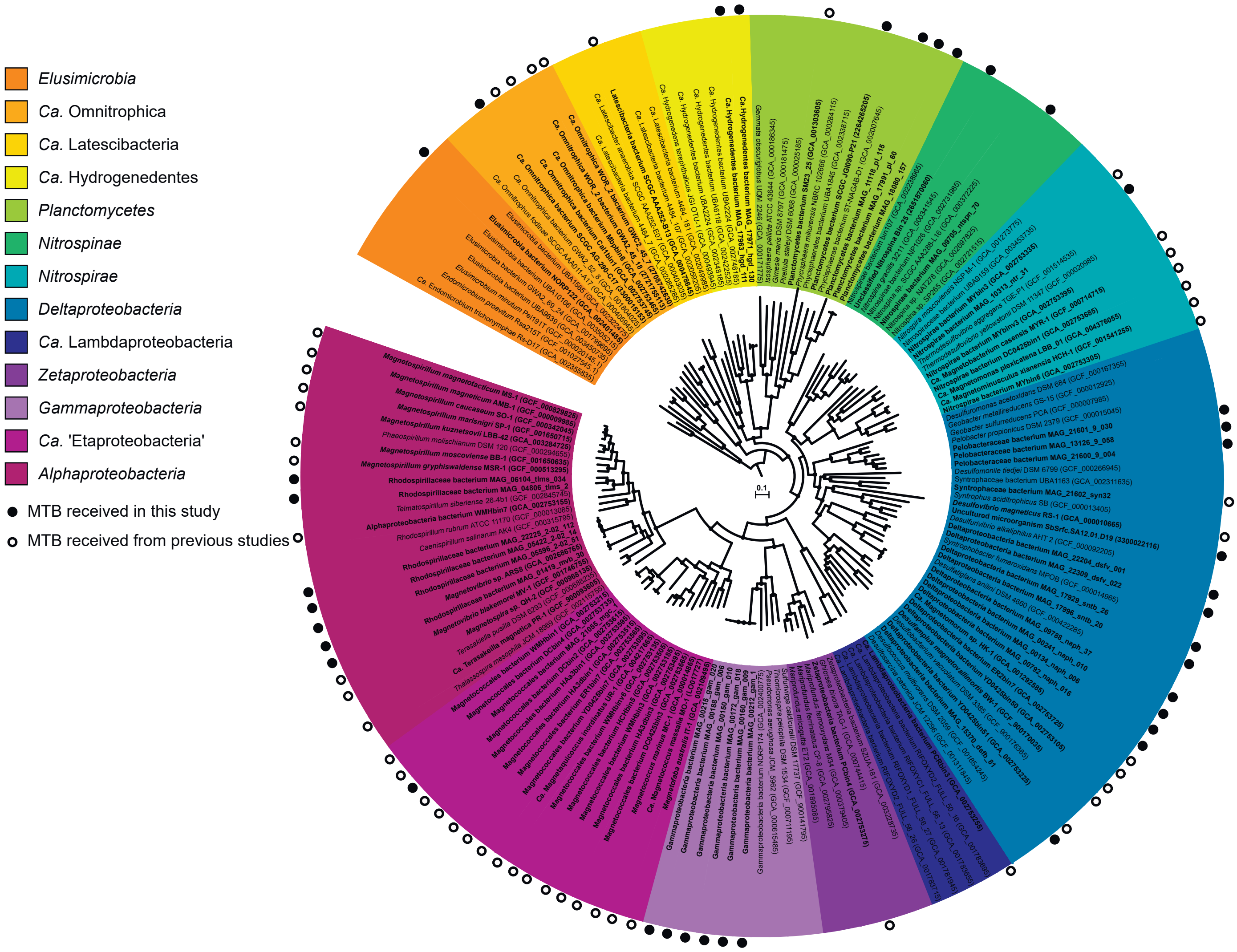

Supplementary Figure S2. Maximum-likelihood phylogenetic tree based on 120 concatenated single copy marker proteins. The tree was reconstructed with the evolutionary model LG+F+I+G4 and 1000 ultrafast bootstraps (bold circles indicate bootstrap 100%). The scale bar represents amino acid substitutions per site.

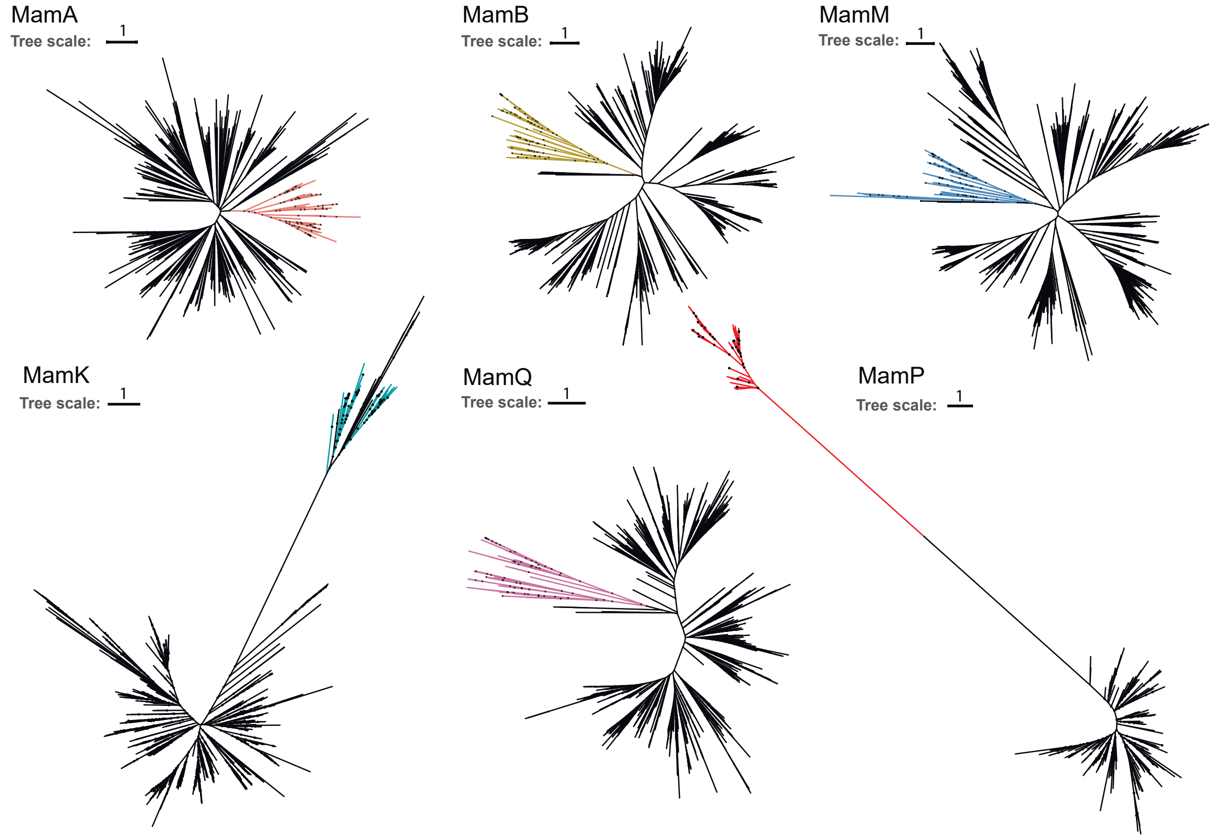

Supplementary Figure S3. Maximum-likelihood phylogenetic trees of MamA, -B, -M, -K, -P, and -Q protein sequences (colored) with their homologs (black). The trees were reconstructed with the evolutionary model LG+F+I+G4 and 1000 ultrafast bootstraps. The scale bar represents amino acid substitutions per site.

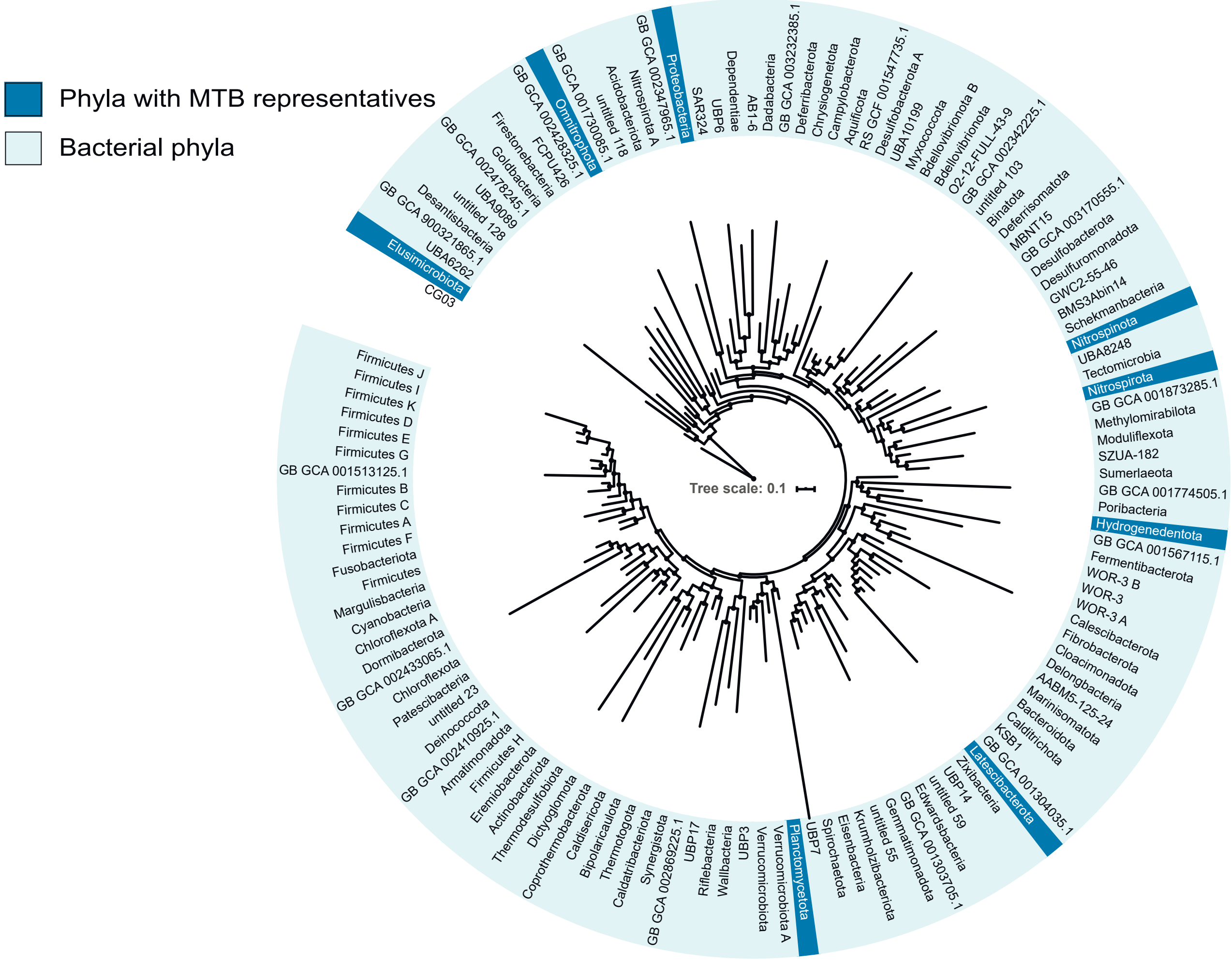

Supplementary Figure S4. The bacterial tree of life, based on GTDB R04-RS89 reference data. The tree was visualised using newick file from annotree site (http://annotree.uwaterloo.ca/app/)

Organism	Phylum/Class	Accession in NCBI/IMG	Taxonomy (GTDB)	Size (bp)	Scaffolds (no.)	GC (%)	N50 (bp)	CheckM completeness (%)	CheckM contamination (%)
Alphaproteobacteria bacterium WMHbin7	*Alphaproteobacteria*	GCA_002753155.1	d__Bacteria;p__Proteobacteria;c__Alphaproteobacteria; o__Rhodospirillales_B;f__WMHbin7; g__WMHbin7;s__WMHbin7 sp002753155	2984788	73	59.84	91589	91.54	0.12
Magnetospira sp. QH-2	*Alphaproteobacteria*	2627853728	d__Bacteria;p__Proteobacteria;c__Alphaproteobacteria; o__Rhodospirillales_A;f__Magnetospiraceae; g__Magnetospira;s__Magnetospira sp000968135	4052370	2	59.44	4021307	99.50	1.00
Magnetospirillum kuznetsovii LBB-42	*Alphaproteobacteria*	GCA_003284725.1	d__Bacteria;p__Proteobacteria;c__Alphaproteobacteria; o__Rhodospirillales_B;f__Magnetospirillaceae; g__Magnetospirillum_A;s__Magnetospirillum_A sp003284725	4405793	69	63.44	215319	99.50	0.50
Magnetospirillum magneticum AMB-1	*Alphaproteobacteria*	637000155	d__Bacteria;p__Proteobacteria;c__Alphaproteobacteria; o__Rhodospirillales_B;f__Magnetospirillaceae; g__Magnetospirillum_A;s__Magnetospirillum_A magneticum	4967148	1	65.09	4967148	99.00	0.00
Magnetospirillum magnetotacticum MS-1	*Alphaproteobacteria*	2645727833	d__Bacteria;p__Proteobacteria;c__Alphaproteobacteria; o__Rhodospirillales_B;f__Magnetospirillaceae; g__Magnetospirillum_A;s__Magnetospirillum_A magnetotacticum	4523935	36	63.56	541957	99.50	0.00
Magnetospirillum marisnigri SP-1	*Alphaproteobacteria*	GCF_001650715.1	d__Bacteria;p__Proteobacteria;c__Alphaproteobacteria; o__Rhodospirillales_B;f__Magnetospirillaceae; g__Magnetospirillum_A;s__Magnetospirillum_A marisnigri	4619819	131	64.73	168256	100.00	0.00
Magnetospirillum moscoviense BB-1	*Alphaproteobacteria*	GCF_001650635.1	d__Bacteria;p__Proteobacteria;c__Alphaproteobacteria ;o__Rhodospirillales_B;f__Magnetospirillaceae; g__Magnetospirillum;s__Magnetospirillum moscoviense	4164497	207	65.18	52974	100.00	0.00
Magnetospirillum sp. ME-1	*Alphaproteobacteria*	GCA_002105535.1	d__Bacteria;p__Proteobacteria;c__Alphaproteobacteria; o__Rhodospirillales;f__Magnetospirillaceae; g__Magnetospirillum_A;s__GCF_002105535.1	4551873	1	65.63	4551873	99.50	0.50
Magnetospirillum sp. XM-1	*Alphaproteobacteria*	GCA_001511835.1	d__Bacteria;p__Proteobacteria;c__Alphaproteobacteria; o__Rhodospirillales;f__Magnetospirillaceae; g__Magnetospirillum_A;s__GCF_001511835.1	4992477	2	65.67	4825187	99.50	0.50
Magnetospirillum caucaseum SO-1	*Alphaproteobacteria*	GCF_000342045.1	d__Bacteria;p__Proteobacteria;c__Alphaproteobacteria; o__Rhodospirillales_B;f__Magnetospirillaceae; g__Magnetospirillum_A;s__Magnetospirillum_A	4870514	236	65.99	55386	99.50	0.50

			caucaseum						
Magnetospirillum gryphiswaldense MSR-1	*Alphaproteobacteria*	GCF_000513295.1	d__Bacteria;p__Proteobacteria;c__Alphaproteobacteria; o__Rhodospirillales_B;f__Magnetospirillaceae; g__Magnetospirillum;s__Magnetospirillum gryphiswaldense	4365716	1	63.29	4365716	99.88	0.00
Magnetovibrio blakemorei MV-1	*Alphaproteobacteria*	GCF_001746755.1	d__Bacteria;p__Proteobacteria;c__Alphaproteobacteria; o__Rhodospirillales_A;f__Magnetovibrionaceae; g__Magnetovibrio;s__Magnetovibrio blakemorei	3638804	91	54.29	95462	98.51	0.50
Terasakiella magnetica PR1	*Alphaproteobacteria*	GCF_900093605.1	d__Bacteria;p__Proteobacteria;c__Alphaproteobacteria; o__Rhodospirillales_A;f__Terasakiellaceae; g__Terasakiella;s__Terasakiella sp900093605	3687561	48	45.97	253666	100.00	0.62
Magnetococcales bacterium DCbin2	*Ca.* Etaproteobacteria	GCA_002753615.1	d__Bacteria;p__Proteobacteria;c__Magnetococcia; o__Magnetococcales;f__UBA8363; g__UBA8363;s__UBA8363 sp002753615	3364396	201	51.85	24837	77.26	1.68
Magnetococcales bacterium DCbin4	*Ca.* Etaproteobacteria	GCA_002753735.1	d__Bacteria;p__Proteobacteria;c__Magnetococcia; o__Magnetococcales;f__UBA8363; g__UBA8363;s__UBA8363 sp002753735	4521331	148	54.25	49732	94.12	2.10
Magnetococcales bacterium ER1bin7	*Ca.* Etaproteobacteria	GCA_002753565.1	d__Bacteria;p__Proteobacteria;c__Magnetococcia; o__Magnetococcales;f__UBA8363; g__GCA-2753565;s__GCA-2753565 sp002753565	3866744	92	52.30	116043	99.11	1.68
Magnetococcales bacterium HA3dbin1	*Ca.* Etaproteobacteria	GCA_002753515.1	d__Bacteria;p__Proteobacteria;c__Magnetococcia; o__Magnetococcales;f__UBA8363; g__UBA8363;s__UBA8363 sp002753515	4326896	134	53.32	54627	96.57	3.36
Magnetococcales bacterium HA3dbin3	*Ca.* Etaproteobacteria	GCA_002753495.1	d__Bacteria;p__Proteobacteria;c__Magnetococcia; o__Magnetococcales;f__DC0425bin3; g__HA3dbin3;s__HA3dbin3 sp002753495	2895658	316	61.72	13187	74.36	2.52
Magnetococcales bacterium HAa3bin1	*Ca.* Etaproteobacteria	GCA_002753595.1	d__Bacteria;p__Proteobacteria;c__Magnetococcia; o__Magnetococcales;f__UBA8363; g__UBA8363;s__UBA8363 sp002753515	4347394	118	53.21	63208	98.25	4.20
Magnetococcales bacterium HCHbin5	*Ca.* Etaproteobacteria	GCA_002753505.1	d__Bacteria;p__Proteobacteria;c__Magnetococcia; o__Magnetococcales;f__WMHbin3; g__HCHbin5;s__HCHbin5 sp002753505	4188618	200	56.97	38237	98.99	4.73
Magnetococcales bacterium WMHbin1	*Ca.* Etaproteobacteria	GCA_002753215.1	d__Bacteria;p__Proteobacteria;c__Magnetococcia; o__Magnetococcales;f__UBA8363; g__UBA8363;s__UBA8363 sp002753735	4380192	242	54.30	24882	98.32	2.94

Magnetococcales bacterium WMHbin3	*Ca.* Etaproteobacteria	GCA_002753185.1	d__Bacteria;p__Proteobacteria;c__Magnetococcia; o__Magnetococcales;f__WMHbin3; g__WMHbin3;s__WMHbin3 sp002753185	4601373	157	61.61	51212	93.53	2.10
Magnetococcales bacterium WMHbinv6	*Ca.* Etaproteobacteria	GCA_002753135.1	d__Bacteria;p__Proteobacteria;c__Magnetococcia; o__Magnetococcales;f__WMHbin3; g__WMHbinv6;s__WMHbinv6 sp002753135	3839927	80	55.32	85378	95.80	2.52
Magnetococcales bacterium YD0425bin7	*Ca.* Etaproteobacteria	GCA_002753095.1	d__Bacteria;p__Proteobacteria;c__Magnetococcia; o__Magnetococcales;f__WMHbin3; g__WMHbinv6;s__WMHbinv6 sp002753095	3583729	177	55.67	34600	96.42	1.68
Magnetococcus marinus MC-1	*Ca.* Etaproteobacteria	639633036	d__Bacteria;p__Proteobacteria;c__Magnetococcia; o__Magnetococcales;f__Magnetococcaceae; g__Magnetococcus;s__Magnetococcus marinus	4719581	1	54.17	4719581	98.32	0.84
Magnetococcus massalia MO-1	*Ca.* Etaproteobacteria	LO017727.1	d__Bacteria;p__Proteobacteria;c__Magnetococcia; o__Magnetococcales;f__Magnetococcaceae; g__;s__	5043095	1	55.18	5043095	98.32	0.00
Magnetofaba australis IT-1	*Ca.* Etaproteobacteria	2786546855	d__Bacteria;p__Proteobacteria;c__Magnetococcia; o__Magnetococcales;f__Magnetococcaceae; g__Magnetofaba;s__Magnetofaba australis	4986701	21	61.30	705613	98.74	0.84
Ca. Magnetaquicoccus inordinatus UR-1	*Ca.* Etaproteobacteria	GCA_004217665.1	d__Bacteria;p__Proteobacteria;c__Magnetococcia; o__Magnetococcales;f__WMHbin3; g__WMHbinv6;s__	4143644	546	52.51	13993	97.40	3.78
Magnetococcales bacterium DC0425bin3	*Ca.* Etaproteobacteria	GCA_002753665.1	d__Bacteria;p__Proteobacteria;c__Magnetococcia; o__Magnetococcales;f__DC0425bin3; g__DC0425bin3;s__DC0425bin3 sp002753665	3696616	230	65.40	26687	98.32	1.68
Ca. Magnetoglobus multicellularis str. Araruama	*Deltaproteobacteria*	2558860350	d__Bacteria;p__Desulfobacterota;c__Desulfobacteria; o__Desulfobacterales;f__Magnetomoraceae; g__;s__	12453848	3705	37.27	6143	98.21	19.24
Ca. Magnetomorum sp. HK-1	*Deltaproteobacteria*	2648501189	d__Bacteria;p__Desulfobacterota;c__Desulfobacteria; o__Desulfobacterales;f__Magnetomoraceae; g__Magnetomorum;s__	14290418	3036	34.61	18233	96.94	3.57
Deltaproteobacteria bacterium ER2bin7	*Deltaproteobacteria*	GCA_002753725.1	d__Bacteria;p__Desulfobacterota;c__Desulfobacteria; o__Desulfobacterales;f__Magnetomoraceae; g__Magnetomorum;s__Magnetomorum sp002753725	5965504	653	38.47	12662	88.06	0.70
Deltaproteobacteria bacterium YD0425bin50	*Deltaproteobacteria*	GCA_002753105.1	d__Bacteria;p__Desulfobacterota;c__Desulfobacteria; o__Desulfobacterales;f__YD0425bin50;	4970345	589	36.91	11398	80.69	2.26

			g__YD0425bin50;s__YD0425bin50 sp002753105						
Deltaproteobacteria bacterium YD0425bin51	*Deltaproteobacteria*	GCA_002753225.1	d__Bacteria;p__Desulfobacterota;c__Desulfobacteria; o__Desulfobacterales;f__YD0425bin51; g__YD0425bin51;s__YD0425bin51 sp002753225	5221616	134	32.23	82896	96.13	0.84
Deltaproteobacterium contig G-2*	*Deltaproteobacteria*	-							
Deltaproteobacterium FH-1*	*Deltaproteobacteria*	-							
Deltaproteobacterium ML-1*	*Deltaproteobacteria*	-							
Desulfamplus magnetovallimortis BW-1	*Deltaproteobacteria*	GCF_900170035.1	d__Bacteria;p__Desulfobacterota;c__Desulfobacteria; o__Desulfobacterales;f__Desulfobacteraceae; g__Desulfamplus;s__Desulfamplus magnetovallimortis	6677998	108	40.72	274785	95.97	2.15
Desulfovibrio magneticus RS-1	*Deltaproteobacteria*	644736352	d__Bacteria;p__Desulfobacterota_A;c__Desulfovibrionia; o__Desulfovibrionales;f__Desulfovibrionaceae; g__Desulfovibrio_F;s__Desulfovibrio_F magneticus	5315620	3	62.67	5248049	100.00	0.00
Gammaproteobacterium Contig I-1*	*Gammaproteobacteria*	3300000174	-						
Gammaproteobacterium Contig I-2*	*Gammaproteobacteria*	3300003600	-						
Gammaproteobacterium SS-5*	*Gammaproteobacteria*	taxonID: 947516	-						
Lambdaproteobacteria bacterium PCRbin3	*Lambdaproteobacteria*	GCA_002753255.1	d__Bacteria;p__SAR324;c__SAR324;o__SAR324; f__GCA-2753255;g__GCA-2753255; s__GCA-2753255 sp002753255	5059199	530	41.83	14906	77.14	0.96
Zetaproteobacteria bacterium PCbin4	*Zetaproteobacteria*	GCA_002753275.1	d__Bacteria;p__Proteobacteria;c__Zetaproteobacteria; o__Mariprofundales;f__Mariprofundaceae; g__GCA-2753275;s__GCA-2753275 sp002753275	1864480	55	47.58	58094	95.33	0.84
Planctomycetes bacterium SM23_25	*Planctomycetes*	2654588058	d__Bacteria;p__Planctomycetota;c__FEN-1346; o__FEN-1346;f__FEN-1346;g__;s__	3770408	418	66.68	9069	62.78	3.98
Ca. Magnetobacterium bavaricum TM-1	*Nitrospirae*	2639762806	d__Bacteria;p__Nitrospirota;c__Thermodesulfovibrionia; o__Thermodesulfovibrionales; f__Magnetobacteriaceae;g__Magnetobacterium;s__	6087939	2147	47.36	4856	94.09	34.50

Ca. Magnetobacterium casensis MYR-1	*Nitrospirae*	2585427832	d__Bacteria;p__Nitrospirota;c__Thermodesulfovibrionia; o__Thermodesulfovibrionales;f__Magnetobacteriaceae; g__Magnetobacterium;s__Magnetobacterium casensis	3415676	70	48.87	90253	92.42	0.91
Ca. Magnetominusculus xianensis HCH-1	*Nitrospirae*	2728369050	d__Bacteria;p__Nitrospirota;c__Thermodesulfovibrionia; o__Thermodesulfovibrionales;f__Magnetobacteriaceae; g__HCH-1;s__HCH-1 sp001541255	3593273	152	45.37	45767	98.18	0.91
Ca. Magnetoovum chiemensis CS-04	*Nitrospirae*	2634166494	d__Bacteria;p__Nitrospirota;c__Thermodesulfovibrionia; o__Thermodesulfovibrionales;f__;g__;s__	3757890	869	40.38	9981	89.01	17.18
Nitrospirae bacterium contig I-3*	*Nitrospirae*	3300000229	-						
Nitrospirae bacterium DC0425bin1	*Nitrospirae*	GCA_002753685.1	d__Bacteria;p__Nitrospirota;c__Thermodesulfovibrionia; o__Thermodesulfovibrionales;f__Magnetobacteriaceae; g__Magnetobacterium; s__Magnetobacterium sp002753685	4024796	107	49.04	75024	96.97	1.82
Nitrospirae bacterium MYbin3	*Nitrospirae*	GCA_002753335.1	d__Bacteria;p__Nitrospirota;c__Thermodesulfovibrionia; o__Thermodesulfovibrionales;f__UBA9935; g__MYbin3;s__MYbin3 sp002753335	2929370	66	44.36	86724	96.49	0.91
Nitrospirae bacterium MYbin6	*Nitrospirae*	GCA_002753305.1	d__Bacteria;p__Nitrospirota;c__Thermodesulfovibrionia; o__Thermodesulfovibrionales; f__Magnetobacteriaceae;g__HCH-1; s__HCH-1 sp002753305	3596763	218	47.78	26590	85.35	2.73
Nitrospirae bacterium MY-3*	*Nitrospirae*	-							
Nitrospirae bacterium MYbin2*	*Nitrospirae*	-							
Nitrospirae bacterium MYbinv3	*Nitrospirae*	GCA_002753395.1	d__Bacteria;p__Nitrospirota;c__Thermodesulfovibrionia; o__Thermodesulfovibrionales; f__Magnetobacteriaceae;g__Magnetobacterium; s__Magnetobacterium sp002753395	3713224	175	44.44	35197	94.24	3.18
Nitrospirae bacterium MY-22*	*Nitrospirae*	-							
Nitrospirae bacterium MY-23*	*Nitrospirae*	-							
Nitrospirae bacterium MY-2*	*Nitrospirae*	-							
Ca.Magnetomonas	*Nitrospirae*	GCA_004376055.1	d__Bacteria;p__Nitrospirota;c__Thermodesulfovibrionia;	2314299	1154	42.13	3183	65.15	1.82

plexicatena LBB_01			o__Thermodesulfovibrionales; f__Magnetobacteriaceae;g__;s__						
Ca. Omnitrophica bacterium WOR_2 GWA2_45_18	*Ca.* Omnitrophica	2721755113	d__Bacteria;p__Omnitrophota;c__koll11;o__UBA10015; f__kpj58rc;g__UBA10174;s__UBA10174 sp003528115	2335743	24	46.36	133212	83.99	1.08
Ca. Omnitrophica bacterium Cal1bin1	*Ca.* Omnitrophica	GCA_002753745.1	d__Bacteria;p__Omnitrophota;c__koll11;o__UBA10015; f__GCA-002753745;g__GCA-2753745; s__GCA-2753745 sp002753745	2540141	240	49.55	15133	84.26	2.15
Ca. Omnitrophica bacterium MBPbin6	*Ca.* Omnitrophica	GCA_002753465.1	d__Bacteria;p__Omnitrophota;c__koll11;o__UBA10015; f__GCA-002753745;g__GCA-2753465; s__GCA-2753465 sp002753465	2181257	150	49.49	24179	68.40	2.15
Ca. Omnitrophica bacterium WOR_2 GWC2_45_7	*Ca.* Omnitrophica	2708742630	d__Bacteria;p__Omnitrophota;c__koll11;o__UBA10015; f__kpj58rc;g__UBA10174;s__	1236942	1352	45.74	1248	56.57	0.10
Ca. Omnitrophus magneticus SKK-01	*Ca.* Omnitrophica	2636416185	d__Bacteria;p__Omnitrophota;c__koll11;o__UBA1560; f__;g__;s__	3103091	529	35.71	15515	96.17	46.77
Latescibacteria bacterium SCGC AAA252-B13	*Ca.* Latescibacteria	2264867252	d__Bacteria;p__Latescibacterota;c__Latescibacteria; o__Latescibacterales;f__Latescibacteraceae;g__;s_	1759976	138	40.86	23078	43.99	0.00
Syntrophaceae bacterium MAG_21602_syn32	*Deltaproteobacteria*	3300021602	d__Bacteria;p__Desulfobacterota;c__Syntrophia; o__Syntrophales;f__;g__;s__	1798526	388	49.78	4890	38.98	8.48

* For these organisms only MGC genes information exists

Supplementary Table S1. Characteristics of MTB genomes used to create the MGC protein sequence database

Organism	Taxonomy (GTDB)
Ca. Hydrogenedentes bacterium MAG_17963_hgd_111	d__Bacteria;p__Hydrogenedentota;c__Hydrogenedentia;o__Hydrogenedentiales;f__;g__;s__
Ca. Hydrogenedentes bacterium MAG_17971_hgd_130	d__Bacteria;p__Hydrogenedentota;c__Hydrogenedentia;o__Hydrogenedentiales;f__;g__;s__
Deltaproteobacteria bacterium MAG_00134_naph_006	d__Bacteria;p__Desulfobacterota;c__Desulfobacteria;o__Desulfatiglandales;f__NaphS2;g__;s__
Deltaproteobacteria bacterium MAG_00241_naph_010	d__Bacteria;p__Desulfobacterota;c__Desulfobacteria;o__Desulfatiglandales;f__NaphS2;g__;s__
Deltaproteobacteria bacterium MAG_00792_naph_016	d__Bacteria;p__Desulfobacterota;c__Desulfobacteria;o__Desulfatiglandales;f__NaphS2;g__;s__
Deltaproteobacteria bacterium MAG_09788_naph_37	d__Bacteria;p__Desulfobacterota;c__Desulfobacteria;o__Desulfatiglandales;f__NaphS2;g__;s__
Deltaproteobacteria bacterium MAG_15370_dsfb_81	d__Bacteria;p__Desulfobacterota;c__Desulfobacteria;o__Desulfobacterales;f__4572-123;g__;s__
Deltaproteobacteria bacterium MAG_17929_sntb_26	d__Bacteria;p__Desulfobacterota;c__Syntrophobacteria;o__Syntrophobacterales;f__Syntrophobacteraceae;g__SbD1;s__
Deltaproteobacteria bacterium MAG_17996_sntb_20	d__Bacteria;p__Desulfobacterota;c__Syntrophobacteria;o__Syntrophobacterales;f__Syntrophobacteraceae;g__SbD1;s__
Deltaproteobacteria bacterium MAG_22204_dsfv_001	d__Bacteria;p__Desulfobacterota;c__Desulfobulbia;o__Desulfobulbales;f__Desulfurivibrionaceae;g__;s_
Deltaproteobacteria bacterium MAG_22309_dsfv_022	d__Bacteria;p__Desulfobacterota;c__Desulfobulbia;o__Desulfobulbales;f__Desulfurivibrionaceae; g__UBA2262;s__
Gammaproteobacteria bacterium MAG_00150_gam_010	d__Bacteria;p__Proteobacteria;c__Gammaproteobacteria;o__GCA-2400775;f__;g__;s__
Gammaproteobacteria bacterium MAG_00160_gam_009	d__Bacteria;p__Proteobacteria;c__Gammaproteobacteria;o__GCA-2400775;f__;g__;s__
Gammaproteobacteria bacterium MAG_00172_gam_018	d__Bacteria;p__Proteobacteria;c__Gammaproteobacteria;o__GCA-2400775;f__;g__;s__
Gammaproteobacteria bacterium MAG_00188_gam_006	d__Bacteria;p__Proteobacteria;c__Gammaproteobacteria;o__GCA-2400775;f__;g__;s__
Gammaproteobacteria bacterium MAG_00212_gam_1	d__Bacteria;p__Proteobacteria;c__Gammaproteobacteria;o__GCA-2400775;f__;g__;s__
Gammaproteobacteria bacterium MAG_00215_gam_020	d__Bacteria;p__Proteobacteria;c__Gammaproteobacteria;o__GCA-2400775;f__;g__;s__
Magnetococcales bacterium MAG_21055_mgc_1	d__Bacteria;p__Proteobacteria;c__Magnetococcia;o__Magnetococcales;f__UBA8363;g__UBA8363;s__
Nitrospinae bacterium MAG_09705_ntspn_70	d__Bacteria;p__Nitrospinota;c__Nitrospinia;o__Nitrospinales;f__Nitrospinaceae;g__UBA8687;s__
Nitrospirae bacterium MAG_10313_ntr_31	d__Bacteria;p__Nitrospirota;c__Thermodesulfovibrionia;o__Thermodesulfovibrionales;f__;g__;s__
Pelobacteraceae bacterium MAG_21601_9_030	d__Bacteria;p__Desulfuromonadota;c__Desulfuromonadia;o__Geobacterales;f__Pelobacteraceae; g__UBA8499;s__

Pelobacteraceae bacterium MAG_13126_9_058	d__Bacteria;p__Desulfuromonadota;c__Desulfuromonadia;o__Geobacterales;f__Pelobacteraceae; g__UBA8499;s__
Pelobacteraceae bacterium MAG_21600_9_004	d__Bacteria;p__Desulfuromonadota;c__Desulfuromonadia;o__Geobacterales;f__Pelobacteraceae; g__UBA8499;s__
Planctomycetes bacterium MAG_11118_pl_115	d__Bacteria;p__Planctomycetota;c__Phycisphaerae;o__SG8-4;f__SG8-4;g__SG8-4;s__
Planctomycetes bacterium MAG_17991_pl_60	d__Bacteria;p__Planctomycetota;c__Phycisphaerae;o__SG8-4;f__SG8-4;g__;s__
Planctomycetes bacterium MAG_18080_pl_157	d__Bacteria;p__Planctomycetota;c__Phycisphaerae;o__SG8-4;f__SG8-4;g__;s__
Rhodospirillaceae bacterium MAG_01419_mvb_30	d__Bacteria;p__Proteobacteria;c__Alphaproteobacteria;o__Rhodospirillales_A;f__Magnetovibrionaceae;g__Magnetovibrio;s__
Rhodospirillaceae bacterium MAG_04806_tlms_2	d__Bacteria;p__Proteobacteria;c__Alphaproteobacteria;o__Rhodospirillales_B;f__Magnetospirillaceae; g__Telmatospirillum;s__
Rhodospirillaceae bacterium MAG_05422_2-02_14	d__Bacteria;p__Proteobacteria;c__Alphaproteobacteria;o__Rhodospirillales_A; f__2-02-FULL-58-16;g__GCA-2686765;s__GCA-2686765 sp002686765
Rhodospirillaceae bacterium MAG_05596_2-02_51	d__Bacteria;p__Proteobacteria;c__Alphaproteobacteria;o__Rhodospirillales_A; f__2-02-FULL-58-16;g__GCA-2686765;s__GCA-2686765 sp002686765
Rhodospirillaceae bacterium MAG_06104_tlms_034	d__Bacteria;p__Proteobacteria;c__Alphaproteobacteria;o__Rhodospirillales_B; f__Magnetospirillaceae;g__;s__
Rhodospirillaceae bacterium MAG_22225_2-02_112	d__Bacteria;p__Proteobacteria;c__Alphaproteobacteria;o__Rhodospirillales_A; f__2-02-FULL-58-16;g__GCA-2686765;s__GCA-2686765 sp002686765
Ca. Omnitrophica bacterium SCGC AG-290-C17	d__Bacteria;p__Omnitrophota;c__Omnitrophia;o__Omnitrophales;f__GWA2-52-8;g__;s__
Magnetovibrio sp. ARS8	d__Bacteria;p__Proteobacteria;c__Alphaproteobacteria;o__Rhodospirillales_A; f__2-02-FULL-58-16;g__GCA-2686765;s__GCA-2686765 sp002686765
Elusimicrobia bacterium NORP122	d__Bacteria;p__Elusimicrobiota;c__Elusimicrobia;o__UBA1565;f__UBA1565; g__UBA1565;s__UBA1565 sp002401485
Uncultured microorganism SbSrfc.SA12.01.D19	d__Bacteria;p__Desulfobacterota;c__Desulfobulbia;o__Desulfobulbales;f__YD12-FULL-57-12;g__;s__
Unclassified Nitrospina Bin 25	d__Bacteria;p__Nitrospinota;c__Nitrospinia;o__Nitrospinales;f__;g__;s__
Planctomycetes bacterium SCGC JGI090-P21	d__Bacteria;p__Planctomycetota;c__Phycisphaerae;o__UBA1845;f__;g__;s__

* For these organisms only MGC genes information exists

Supplementary Table S2. Taxonomic assignments obtained from the Genome Taxonomy Database for the MAGS, SAGs, and genomes obtained in this study.

IMG metagenome ID	Sampling place	Binned in this work	Number of obtained MAGs
3300000124	Marine microbial communities from chronically polluted sediments in the Baltic Sea - site KBA sample SWE 12_21m	+	85
3300000126	Marine microbial communities from chronically polluted sediments in the Baltic Sea - site KBB sample SWE 26_20.5m	+	63
3300000134	Marine microbial communities from chronically polluted sediments in the Baltic Sea - site KBA sample SWE 07_21m	+	36
3300000150	Marine microbial communities from expanding oxygen minimum zones in the Saanich Inlet - 48 08/11/10 120m	+	53
3300000151	Marine microbial communities from expanding oxygen minimum zones in the Saanich Inlet - 53 01/11/11 200m	+	62
3300000154	Marine microbial communities from expanding oxygen minimum zones in the Saanich Inlet - 47 07/07/10 150m	+	79
3300000160	Marine microbial communities from expanding oxygen minimum zones in the Saanich Inlet - 48 08/11/10 135m	+	55
3300000164	Marine microbial communities from expanding oxygen minimum zones in the Saanich Inlet - 39 11/10/09 200m	+	93
3300000172	Marine microbial communities from expanding oxygen minimum zones in the Saanich Inlet - 34 06/16/09 200m	+	80
3300000188	Marine microbial communities from expanding oxygen minimum zones in the Saanich Inlet - 60 08/10/11 150m	+	38
3300000193	Marine microbial communities from expanding oxygen minimum zones in the Saanich Inlet - 47 07/07/10 135m	+	72
3300000201	Marine microbial communities from expanding oxygen minimum zones in the Saanich Inlet - 54 02/08/11 135m	+	39
3300000211	Marine microbial communities from expanding oxygen minimum zones in the Saanich Inlet - 53 01/11/11 135m	+	56
3300000212	Marine microbial communities from expanding oxygen minimum zones in the Saanich Inlet - 47 07/07/10 120m	+	92
3300000213	Marine microbial communities from expanding oxygen minimum zones in Line P, North Pacific Ocean - sample_F_10_SI03_150	+	66
3300000214	Marine microbial communities from expanding oxygen minimum zones in the Saanich Inlet - 54 02/08/11 200m	+	46
3300000215	Marine microbial communities from expanding oxygen minimum zones in the Saanich Inlet - 53 01/11/11 120m	+	75
3300000216	Marine microbial communities from expanding oxygen minimum zones in the Saanich Inlet - 53 01/11/11 150m	+	87
3300000229	Groundwater microbial communities from subsurface biofilms in sulfidic aquifier in Frasassi Gorge, Italy, sample from two redox zones-LI09_3	+	39
3300000241	Marine microbial communities from chronically polluted sediments in the Baltic Sea - site KBB sample SWE 21_20.5m	+	74
3300000792	Marine microbial communities from chronically polluted sediments in the Baltic Sea - site KBA sample SWE 02_21m	+	119
3300001419	Saline surface water microbial communities from Etoliko Lagoon, Greece - halocline water (15 m)	+	113
3300001687	Deep Marine Sediments WOR-3-8_10	+	147
3300001751	Marine sediment microbial communities from White Oak River estuary, North Carolina - WOR-2-30_32	+	88

3300001752	Marine sediment microbial communities from White Oak River estuary, North Carolina - WOR-1-36_30	-*	0
3300001753	Marine sediment microbial communities from White Oak River estuary, North Carolina - WOR-3-24_28	+	173
3300001782	Marine sediment microbial communities from White Oak River estuary, North Carolina - WOR_deep_samples	+	172
3300001854	Marine sediment microbial communities from White Oak River estuary, North Carolina - WOR-1-52-54	+	228
3300002053	Marine sediment microbial communities from White Oak River estuary, North Carolina - WOR_SMTZ	+	198
3300002231	Marine sediment microbial communities from Santorini caldera mats, Greece - red mat	+	176
3300002529	Soil microbial communities from Rifle, Colorado - Rifle CSP2_plank highO2_0.2	+	465
3300003144	Marine sediment microbial communities from deep subseafloor - Sample from 18.6 mbsf	+	14
3300003492	Marine microbial communities from expanding oxygen minimum zones in the Saanich Inlet - SI037_S4LV_200m_DNA	-***	0
3300003496	Marine microbial communities from expanding oxygen minimum zones in the Saanich Inlet - SI037_S3LV_200m_DNA	-***	0
3300003582	Marine microbial communities from expanding oxygen minimum zones in the Saanich Inlet - SI073_LV_10m_DNA	-***	0
3300003590	Marine microbial communities from expanding oxygen minimum zones in the Saanich Inlet - SI072_LV_200m_DNA	-***	0
3300003593	Marine microbial communities from expanding oxygen minimum zones in the Saanich Inlet - SI074_LV_100m_DNA	-***	0
3300003594	Marine microbial communities from expanding oxygen minimum zones in the Saanich Inlet - SI074_LV_10m_DNA	-***	0
3300003595	Marine microbial communities from expanding oxygen minimum zones in the Saanich Inlet - SI074_LV_200m_DNA	-***	0
3300003599	Marine microbial communities from expanding oxygen minimum zones in the Saanich Inlet - SI037_S3LV_10m_DNA	-***	0
3300003615	Marine microbial communities from expanding oxygen minimum zones in the Saanich Inlet - SI037_S3LV_110m_DNA	-***	0
3300003894	Marine microbial communities from the northern Gulf of Mexico hypoxic zone - Cultivation independent assessment	+	103
3300004109	Marine microbial communities from expanding oxygen minimum zones in the Saanich Inlet - SI037_S2LV_150m_DNA	-***	0
3300004111	Marine microbial communities from expanding oxygen minimum zones in the Saanich Inlet - SI037_S2LV_200m_DNA	-***	0
3300004273	Marine microbial communities from expanding oxygen minimum zones in the Saanich Inlet - SI075_LV_DNA_135m	-***	0
3300004274	Marine microbial communities from expanding oxygen minimum zones in the Saanich Inlet - SI075_LV_DNA_120m	-***	0
3300004276	Marine microbial communities from expanding oxygen minimum zones in the Saanich Inlet - SI075_LV_DNA_165m	-***	0
3300004277	Marine microbial communities from expanding oxygen minimum zones in the Saanich Inlet - SI075_LV_DNA_200m	-***	0
3300004278	Marine microbial communities from expanding oxygen minimum zones in the Saanich Inlet - SI075_LV_DNA_150m	-***	0
3300004774	Freshwater microbial communities from Crystal Bog, Wisconsin, USA - MA5M	+	64
3300004806	Freshwater microbial communities from Crystal Bog, Wisconsin, USA - CBH12Aug08	+	160
3300005346	Saline sediment microbial community from Etoliko Lagoon, Greece	+	20
3300005408	Marine microbial and viral communities from oxygen minimum zone, Eastern Pacific Ocean - ETNP201310SV72	+	60
3300005422	Marine microbial and viral communities from oxygen minimum zone, Eastern Pacific Ocean - ETNP201306SV43	+	74

3300005551	Marine microbial and viral communities from oxygen minimum zone, Eastern Pacific Ocean - ETNP201302PF89A	+	47
3300005592	Marine microbial and viral communities from oxygen minimum zone, Eastern Pacific Ocean - ETNP201302SV89	+	65
3300005596	Marine microbial and viral communities from oxygen minimum zone, Eastern Pacific Ocean - ETNP201306PF43B	+	63
3300005838	Marine microbial communities from expanding oxygen minimum zones in the Saanich Inlet - SI037_S2LV_130m_DNA	-***	0
3300005919	Saline lake microbial communities from Ace Lake, Antarctica - Antarctic Ace Lake Metagenome 02UKM	+	117
3300005939	Saline lake microbial communities from Ace Lake, Antarctica - Antarctic Ace Lake Metagenome 02UKX	+	101
3300006104	Freshwater microbial communities from Crystal Bog, Wisconsin, USA - CBH12Aug09.1	+	134
3300006113	Freshwater microbial communities from Crystal Bog, Wisconsin, USA - CBH08Aug08	+	35
3300009149	Deep subsurface microbial communities from Baltic Sea to uncover new lineages of life (NeLLi) - Landsort_02402 metaG	+	117
3300009150	Deep subsurface microbial communities from South Atlantic Ocean to uncover new lineages of life (NeLLi) - Benguela_00093 metaG	+	222
3300009175	Freshwater lake bacterial and archeal communities from Alinen Mustajarvi, Finland, to study Microbial Dark Matter (Phase II) - Alinen Mustajarvi 5m metaG	+	251
3300009444	Hot spring microbial communities from Beatty, Nevada to study Microbial Dark Matter (Phase II) - OV2 TP3	+	187
3300009488	Deep subsurface microbial communities from Indian Ocean to uncover new lineages of life (NeLLi) - Sumatra_00607 metaG	+	156
3300009528	Deep subsurface microbial communities from South Pacific Ocean to uncover new lineages of life (NeLLi) - Chile_00310 metaG	+	197
3300009529	Deep subsurface microbial communities from Black Sea to uncover new lineages of life (NeLLi) - Black_00105 metaG	+	205
3300009548	Peatland microbial communities from Minnesota, USA, analyzing carbon cycling and trace gas fluxes - June2015DPH_6_100	+	66
3300009631	Peatland microbial communities from Minnesota, USA, analyzing carbon cycling and trace gas fluxes - June2015DPH_10_100	+	38
3300009691	Hot spring microbial communities from Beatty, Nevada to study Microbial Dark Matter (Phase II) - OV2 TP2	+	127
3300009705	Marine microbial communities from western Arctic Ocean - ArcticOcean_MG_CB8_128	+	219
3300009788	Deep subsurface microbial communities from Indian Ocean to uncover new lineages of life (NeLLi) - Sumatra_00157 metaG	+	192
3300010313	Hot spring microbial communities from South Africa to study Microbial Dark Matter (Phase II) - Sagole hot spring metaG	+	136
3300010341	Bog forest soil microbial communities from Calvert Island, British Columbia, Canada - Bog Forest MetaG ECP23OM2	+	134
3300010883	Western Arctic Ocean co-assembly	- data from 3300009705	0

3300011118	Deep subsurface microbial communities from Aarhus Bay to uncover new lineages of life (NeLLi) - Aarhus_00045 metaG	+	282
3300013089	Freshwater microbial communities from Powell Lake, British Columbia, Canada to study Microbial Dark Matter (Phase II) - PL_2010_330m	+	165
3300013098	Subseafloor sediment microbial communities from Guaymas Basin, Gulf of California, Mexico - Guay11, Core 4567-28, 0-3 cm	+	87
3300013101	Subseafloor sediment microbial communities from Guaymas Basin, Gulf of California, Mexico - Guay4, Core 4569-4, 0-3 cm	+	208
3300013126	Freshwater microbial communities from Kabuno Bay, South-Kivu, Congo ? kab_022012_10m	+	275
3300013131	Freshwater microbial communities from Kabuno Bay, South-Kivu, Congo ? kab_092012_10m	-*	0
3300013232	Sediment microbial communities from Acid Mine Drainage holding pond in Pittsburgh, PA, USA	+	228
3300014151	Peatland microbial communities from Houghton, MN, USA - PEATcosm2014_Bin23_60_metaG	+	82
3300014152	Peatland microbial communities from Houghton, MN, USA - PEATcosm2014_Bin11_60_metaG	+	96
3300014153	Peatland microbial communities from Houghton, MN, USA - PEATcosm2014_Bin06_60_metaG	+	92
3300014158	Peatland microbial communities from Houghton, MN, USA - PEATcosm2014_Bin02_60_metaG	-*	0
3300014159	Peatland microbial communities from Houghton, MN, USA - PEATcosm2014_Bin10_60_metaG	+	113
3300014491	Permafrost microbial communities from Stordalen Mire, Sweden - 612S2D metaG	+	168
3300014638	Peatland microbial communities from Houghton, MN, USA - PEATcosm2014_Bin17_60_metaG	+	110
3300014654	Peatland microbial communities from Houghton, MN, USA - PEATcosm2014_Bin06_10_metaG	+	149
3300014903	Subseafloor sediment microbial communities from Guaymas Basin, Gulf of California, Mexico - Guay12, Core 4567-28, 21-24 cm	+	134
3300015153	Sorted cell/s from water in Cold Creek, Beatty, Nevada, USA - Omnitrophica bacterium SCGC_AG-290-C17	-**	0
3300015370	Groundwater microbial communities from the Aspo Hard Rock Laboratory (HRL) deep subsurface site, Sweden - OS_PC_MetaG	+	238
3300017818	Coastal salt marsh microbial communities from the Groves Creek Marsh, Skidaway Island, Georgia - 101401AT metaG (megahit assembly)	+	249
3300017925	Peatland microbial communities from SPRUCE experiment site at the Marcell Experimental Forest, Minnesota, USA - June2016WEW_8_40	+	68
3300017929	Peatland microbial communities from SPRUCE experiment site at the Marcell Experimental Forest, Minnesota, USA - June2016WEW_4_100	+	111
3300017963	Hypersaline lake sediment archaeal communities from the Salton Sea, California, USA - SS_3_D_1 metaG	+	170
3300017971	Hypersaline lake sediment archaeal communities from the Salton Sea, California, USA - SS_3_D_2 metaG	+	207
3300017985	Coastal salt marsh microbial communities from the Groves Creek Marsh, Skidaway Island, Georgia - 101412BT metaG (megahit assembly)	+	220

3300017987	Hypersaline lake sediment archaeal communities from the Salton Sea, California, USA - SS_1_MS_1 metaG	+	238
3300017991	Hypersaline lake sediment archaeal communities from the Salton Sea, California, USA - SS_1_D_2 metaG	+	218
3300017996	Peatland microbial communities from SPRUCE experiment site at the Marcell Experimental Forest, Minnesota, USA - June2016WEW_21_40	+	66
3300018002	Peatland microbial communities from SPRUCE experiment site at the Marcell Experimental Forest, Minnesota, USA - June2016WEW_13_40	+	59
3300018004	Peatland microbial communities from SPRUCE experiment site at the Marcell Experimental Forest, Minnesota, USA - June2016WEW_11_100	+	84
3300018016	Peatland microbial communities from SPRUCE experiment site at the Marcell Experimental Forest, Minnesota, USA - June2016WEW_19_40	+	88
3300018019	Peatland microbial communities from SPRUCE experiment site at the Marcell Experimental Forest, Minnesota, USA - June2016WEW_16_150	+	84
3300018023	Peatland microbial communities from SPRUCE experiment site at the Marcell Experimental Forest, Minnesota, USA - June2016WEW_7_100	+	105
3300018024	Peatland microbial communities from SPRUCE experiment site at the Marcell Experimental Forest, Minnesota, USA - June2016WEW_19_100	+	142
3300018025	Peatland microbial communities from SPRUCE experiment site at the Marcell Experimental Forest, Minnesota, USA - June2016WEW_20_100	+	101
3300018026	Peatland microbial communities from SPRUCE experiment site at the Marcell Experimental Forest, Minnesota, USA - June2016WEW_8_100	+	93
3300018033	Peatland microbial communities from SPRUCE experiment site at the Marcell Experimental Forest, Minnesota, USA - June2016WEW_13_10	+	107
3300018080	Hypersaline lake sediment archaeal communities from the Salton Sea, California, USA - SS_1_D_1 metaG	+	270
3300018426	Coastal salt marsh microbial communities from the Groves Creek Marsh, Skidaway Island, Georgia - 101402AT metaG (megahit assembly)	+	250
3300019082	Peatland microbial communities from SPRUCE experiment site at the Marcell Experimental Forest, Minnesota, USA - June2016WEW_6_40	-*	0
3300020158	Anoxic zone freshwater microbial communities from boreal shield lake in IISD Experimental Lakes Area, Ontario, Canada - Jun2016-L227-6m	+	97
3300020164	Anoxic zone freshwater microbial communities from boreal shield lake in IISD Experimental Lakes Area, Ontario, Canada - Jun2016-L304-6m	+	159
3300021055	Subsurface sediment microbial communities from Mancos shale, Colorado, United States - Mancos C3	+	17
3300021070	Anoxic zone freshwater microbial communities from boreal shield lake in IISD Experimental Lakes Area, Ontario, Canada - Sep2016-L442-13m	+	157
3300021071	Anoxic zone freshwater microbial communities from boreal shield lake in IISD Experimental Lakes Area, Ontario, Canada - Sep2016-L442-17m	+	90

3300021072	Anoxic zone freshwater microbial communities from boreal shield lake in IISD Experimental Lakes Area, Ontario, Canada - Sep2016-L442-15m	+	100
3300021074	Anoxic zone freshwater microbial communities from boreal shield lake in IISD Experimental Lakes Area, Ontario, Canada - Jun2016-L442-17m	+	169
3300021075	Anoxic zone freshwater microbial communities from boreal shield lake in IISD Experimental Lakes Area, Ontario, Canada - Sep2016-L373-20m	+	153
3300021354	Anoxic zone freshwater microbial communities from boreal shield lake in IISD Experimental Lakes Area, Ontario, Canada - Jun2016-L221-5m	+	199
3300021600	Anoxic zone freshwater microbial communities from boreal shield lake in IISD Experimental Lakes Area, Ontario, Canada - Sep2016-L626-11m	+	122
3300021601	Anoxic zone freshwater microbial communities from boreal shield lake in IISD Experimental Lakes Area, Ontario, Canada - Sep2016-L224-21m	+	136
3300021602	Anoxic zone freshwater microbial communities from boreal shield lake in IISD Experimental Lakes Area, Ontario, Canada - Sep2016-L222-5m	+	262
3300022116	Sorted cell/s from Aspo Hard Rock Laboratory (HRL) deep subsurface site groundwater, Oskarshamn, Sweden - uncultured microorganism SbSrfc.SA12.01.D19	-**	0
3300022204	Sediment microbial communities from San Francisco Bay, California, United States - SF_Jul11_sed_USGS_8_1	+	187
3300022214	Sediment microbial communities from San Francisco Bay, California, United States - SF_Jan12_sed_USGS_4_1	+	143
3300022217	Sediment microbial communities from San Francisco Bay, California, United States - SF_May12_sed_USGS_24	+	101
3300022223	Sediment microbial communities from San Francisco Bay, California, United States - SF_Oct11_sed_USGS_8_1	+	234
3300022225	Marine microbial and viral communities from oxygen minimum zone, Eastern Pacific Ocean - ETNP2014_SV_400_PacBio MetaG (Illumina Assembly)	+	373
3300022309	Sediment microbial communities from San Francisco Bay, California, United States - SF_May12_sed_USGS_4_1	+	159
3300022553	Powell_combined assembly	- data from 3300013089	0
3300022555	Alinen_combined assembly	- data from 3300009175	0
**Total MAGs**			**14688**
-*	No binning results were obtained		
-**	Sorted cells, no needed to be binned		
-***	Metagenomes were not binned because samples were obtained from one place and contained 100% identical MamK sequences		

Supplementary Table S3. Metagenomes detected to contain *mamK* genes, and the number of MAGs obtained after metagenome binning.

Genome ID	Scaffold ID	Gene_oid	Scaffold Length (bp)	Scaffold GC %	Scaffold Read Depth	Ecosystem Category	Ecosystem Subtype	Ecosystem Type
3300000124	BS_KBA_SWE12_21mDRAFT_c10006010	3300000124 assembled BS_KBA_SWE12_21mDRAFT_100060105	4170	0.41	23	Aquatic	Wetlands	Marine
3300000124	BS_KBA_SWE12_21mDRAFT_c10000022	3300000124 assembled BS_KBA_SWE12_21mDRAFT_1000002221	50993	0.52	47	Aquatic	Wetlands	Marine
3300000126	BS_KBB_SWE26_205mDRAFT_c1003348	3300000126 assembled BS_KBB_SWE26_205mDRAFT_10033483	2958	0.4	39	Aquatic	Wetlands	Marine
3300000126	BS_KBB_SWE26_205mDRAFT_c1016174	3300000126 assembled BS_KBB_SWE26_205mDRAFT_10161741	1238	0.51	43	Aquatic	Wetlands	Marine
3300000134	BS_KBA_SWE07_21mDRAFT_c1000050	3300000134 assembled BS_KBA_SWE07_21mDRAFT_100005014	14693	0.49	24	Aquatic	Wetlands	Marine
3300000150	SI48aug10_120mDRAFT_c1000041	3300000150 assembled SI48aug10_120mDRAFT_100004115	39017	0.48	53	Aquatic	Intertidal zone	Marine
3300000151	SI53jan11_200mDRAFT_c1000626	3300000151 assembled SI53jan11_200mDRAFT_100062617	18423	0.48	128	Aquatic	Intertidal zone	Marine
3300000151	SI53jan11_200mDRAFT_c1001635	3300000151 assembled SI53jan11_200mDRAFT_10016355	9965	0.5	102	Aquatic	Intertidal zone	Marine
3300000154	SI47jul10_150mDRAFT_c1000105	3300000154 assembled SI47jul10_150mDRAFT_100010517	46665	0.48	62	Aquatic	Intertidal zone	Marine
3300000154	SI47jul10_150mDRAFT_c1008647	3300000154 assembled SI47jul10_150mDRAFT_10086472	3064	0.46	42	Aquatic	Intertidal zone	Marine
3300000160	SI48aug10_135mDRAFT_c1000085	3300000160 assembled SI48aug10_135mDRAFT_100008525	40601	0.48	76	Aquatic	Intertidal zone	Marine
3300000164	SI39no09_200mDRAFT_c1002767	3300000164 assembled SI39no09_200mDRAFT_10027676	6943	0.46	42	Aquatic	Intertidal zone	Marine
3300000164	SI39no09_200mDRAFT_c1009836	3300000164 assembled SI39no09_200mDRAFT_10098362	3069	0.46	44	Aquatic	Intertidal zone	Marine
3300000172	SI34jun09_200mDRAFT_c1000137	3300000172 assembled SI34jun09_200mDRAFT_100013717	41737	0.48	93	Aquatic	Intertidal zone	Marine
3300000174	SI60aug11_200mDRAFT_c1000053	3300000174 assembled SI60aug11_200mDRAFT_100005332	69464	0.48	89	Aquatic	Intertidal zone	Marine
3300000174	SI60aug11_200mDRAFT_c1007538	3300000174 assembled SI60aug11_200mDRAFT_10075385	3183	0.46	14	Aquatic	Intertidal zone	Marine

3300000188	SI60aug11_150mDRAFT_c1000208	3300000188 assembled SI60aug11_150mDRAFT_10002087	16603	0.48	82	Aquatic	Intertidal zone	Marine
3300000193	SI47jul10_135mDRAFT_c1000134	3300000193 assembled SI47jul10_135mDRAFT_100013417	41676	0.48	54	Aquatic	Intertidal zone	Marine
3300000201	SI54feb11_135mDRAFT_c1009650	3300000201 assembled SI54feb11_135mDRAFT_10096502	1253	0.46	39	Aquatic	Intertidal zone	Marine
3300000211	SI53jan11_135mDRAFT_c1011890	3300000211 assembled SI53jan11_135mDRAFT_10118902	1525	0.45	15	Aquatic	Intertidal zone	Marine
3300000212	SI47jul10_120mDRAFT_c1002535	3300000212 assembled SI47jul10_120mDRAFT_10025357	6779	0.46	22	Aquatic	Intertidal zone	Marine
3300000213	LP_F_10_SI03_150DRAFT_c1002574	3300000213 assembled LP_F_10_SI03_150DRAFT_10025745	5493	0.46	24	Aquatic	Oceanic	Marine
3300000214	SI54feb11_200mDRAFT_c1000740	3300000214 assembled SI54feb11_200mDRAFT_10007407	10174	0.47	50	Aquatic	Intertidal zone	Marine
3300000214	SI54feb11_200mDRAFT_c1004724	3300000214 assembled SI54feb11_200mDRAFT_10047242	3047	0.46	34	Aquatic	Intertidal zone	Marine
3300000215	SI53jan11_120mDRAFT_c1000197	3300000215 assembled SI53jan11_120mDRAFT_100019717	31918	0.48	34	Aquatic	Intertidal zone	Marine
3300000216	SI53jan11_150mDRAFT_c1000132	3300000216 assembled SI53jan11_150mDRAFT_100013217	46665	0.48	107	Aquatic	Intertidal zone	Marine
3300000216	SI53jan11_150mDRAFT_c1002381	3300000216 assembled SI53jan11_150mDRAFT_10023814	8217	0.5	78	Aquatic	Intertidal zone	Marine
3300000229	TB_LI09_3DRAFT_1003818	3300000229 assembled TB_LI09_3DRAFT_10038186	4036	0.42	90	Aquatic	Groundwater	Freshwater
3300000241	BS_KBA_SWE21_205mDRAFT_10000112	3300000241 assembled BS_KBA_SWE21_205mDRAFT_1000011211	24445	0.51	43	Aquatic	Wetlands	Marine
3300000792	BS_KBA_SWE02_21mDRAFT_10000792	3300000792 assembled BS_KBA_SWE02_21mDRAFT_100007925	11970	0.41	30	Aquatic	Intertidal zone	Marine
3300000792	BS_KBA_SWE02_21mDRAFT_10000057	3300000792 assembled BS_KBA_SWE02_21mDRAFT_1000005719	34622	0.52	57	Aquatic	Intertidal zone	Marine
3300001419	JGI11705J14877_10007165	3300001419 assembled JGI11705J14877_100071654	5184	0.4	24	Aquatic	Saline	Non-marine Saline and Alkaline
3300001419	JGI11705J14877_10001955	3300001419 assembled JGI11705J14877_100019552	11420	0.54	52	Aquatic	Saline	Non-marine Saline and Alkaline

3300001419	JGI11705J14877_10006387	3300001419 assembled JGI11705J14877_100063875	5557	0.55	54	Aquatic	Saline	Non-marine Saline and Alkaline
3300001457	JGI10211J14876_10000097	3300001457 assembled JGI10211J14876_1000009714	31606	0.47	64	Aquatic	Saline	Non-marine Saline and Alkaline
3300001457	JGI10211J14876_10000325	3300001457 assembled JGI10211J14876_1000032526	20233	0.45	61	Aquatic	Saline	Non-marine Saline and Alkaline
3300001457	JGI10211J14876_10001919	3300001457 assembled JGI10211J14876_1000191912	9038	0.43	54	Aquatic	Saline	Non-marine Saline and Alkaline
3300001687	WOR8_10023872	3300001687 assembled WOR8_100238721	6643	0.48	1	Aquatic	Oceanic	Marine
3300001751	JGI2172J19969_10045593	3300001751 assembled JGI2172J19969_100455931	1470	0.5	19	Aquatic	Oceanic	Marine
3300001751	JGI2172J19969_10006683	3300001751 assembled JGI2172J19969_100066832	4899	0.45	34	Aquatic	Oceanic	Marine
3300001752	JGI2173J19968_10033383	3300001752 assembled JGI2173J19968_100333832	1729	0.47	35	Aquatic	Oceanic	Marine
3300001753	JGI2171J19970_10006814	3300001753 assembled JGI2171J19970_100068149	5837	0.45	35	Aquatic	Oceanic	Marine
3300001753	JGI2171J19970_10034696	3300001753 assembled JGI2171J19970_100346962	2081	0.47	18	Aquatic	Oceanic	Marine
3300001782	WOR52_10036001	3300001782 assembled WOR52_100360017	6686	0.4	1	Aquatic	Intertidal zone	Marine
3300001782	WOR52_10005260	3300001782 assembled WOR52_1000526019	18039	0.45	1	Aquatic	Intertidal zone	Marine
3300001854	JGI24422J19971_10215508	3300001854 assembled JGI24422J19971_102155081	823	0.54	20	Aquatic	Oceanic	Marine
3300001854	JGI24422J19971_10010471	3300001854 assembled JGI24422J19971_100104711	5723	0.46	63	Aquatic	Oceanic	Marine
3300001854	JGI24422J19971_10047837	3300001854 assembled JGI24422J19971_100478372	2446	0.47	44	Aquatic	Oceanic	Marine
3300002053	SMTZ23_10026417	3300002053 assembled SMTZ23_100264174	8092	0.48	1	Aquatic	Intertidal zone	Marine
3300002053	SMTZ23_10016270	3300002053 assembled SMTZ23_100162706	10586	0.41	1	Aquatic	Intertidal zone	Marine
3300002053	SMTZ23_10024145	3300002053 assembled SMTZ23_100241451	19124	0.48	1	Aquatic	Intertidal zone	Marine
3300002053	SMTZ23_10053527	3300002053 assembled SMTZ23_100535279	5364	0.47	1	Aquatic	Intertidal zone	Marine
3300002053	SMTZ23_10062872	3300002053 assembled SMTZ23_100628727	10954	0.47	1	Aquatic	Intertidal zone	Marine
3300002231	KVRMV2_100013668	3300002231 assembled KVRMV2_1000136681	2158	0.46	14	Aquatic	Hydrothermal vents	Marine
3300002231	KVRMV2_100490840	3300002231 assembled KVRMV2_1004908401	2681	0.43	7	Aquatic	Hydrothermal vents	Marine
3300002231	KVRMV2_100041118	3300002231 assembled KVRMV2_1000411182	8860	0.48	10	Aquatic	Hydrothermal vents	Marine
3300002231	KVRMV2_100038070	3300002231 assembled KVRMV2_1000380704	16112	0.61	14	Aquatic	Hydrothermal vents	Marine
3300002231	KVRMV2_101240214	3300002231 assembled KVRMV2_1012402141	1445	0.46	7	Aquatic	Hydrothermal vents	Marine

3300002466	JGI10211J34971_10000686	3300002466 assembled JGI10211J34971_1000068612	28102	0.46	89	Aquatic	Saline	Non-marine Saline and Alkaline
3300002466	JGI10211J34971_10000961	3300002466 assembled JGI10211J34971_1000096121	23951	0.54	63	Aquatic	Saline	Non-marine Saline and Alkaline
3300002466	JGI10211J34971_10000961	3300002466 assembled JGI10211J34971_100009615	23951	0.54	63	Aquatic	Saline	Non-marine Saline and Alkaline
3300002466	JGI10211J34971_10001361	3300002466 assembled JGI10211J34971_1000136125	20232	0.45	83	Aquatic	Saline	Non-marine Saline and Alkaline
3300002466	JGI10211J34971_10003099	3300002466 assembled JGI10211J34971_100030994	13387	0.45	70	Aquatic	Saline	Non-marine Saline and Alkaline
3300002466	JGI10211J34971_10011232	3300002466 assembled JGI10211J34971_100112324	6601	0.4	28	Aquatic	Saline	Non-marine Saline and Alkaline
3300002529	C687J35504_10005579	3300002529 assembled C687J35504_1000557911	7848	0.43	25	Terrestrial	Loam	Soil
3300003144	Ga0052244_1005653	3300003144 assembled Ga0052244_10056532	1461	0.45	1	Aquatic	Neritic zone	Marine
3300003492	JGI26245J51145_1000097	3300003492 assembled JGI26245J51145_100009717	56481	0.48	32	Aquatic	Intertidal zone	Marine
3300003492	JGI26245J51145_1000691	3300003492 assembled JGI26245J51145_10006915	19094	0.49	39	Aquatic	Intertidal zone	Marine
3300003496	JGI26241J51128_1000792	3300003496 assembled JGI26241J51128_10007925	19114	0.49	50	Aquatic	Intertidal zone	Marine
3300003496	JGI26241J51128_1000956	3300003496 assembled JGI26241J51128_10009561	16657	0.48	42	Aquatic	Intertidal zone	Marine
3300003582	JGI26252J51714_1000163	3300003582 assembled JGI26252J51714_10001633	44691	0.48	35	Aquatic	Intertidal zone	Marine
3300003582	JGI26252J51714_1004880	3300003582 assembled JGI26252J51714_10048802	4109	0.48	37	Aquatic	Intertidal zone	Marine
3300003590	JGI26251J51716_1000099	3300003590 assembled JGI26251J51716_100009917	43480	0.48	91	Aquatic	Intertidal zone	Marine
3300003590	JGI26251J51716_1000679	3300003590 assembled JGI26251J51716_10006794	9944	0.5	109	Aquatic	Intertidal zone	Marine
3300003593	JGI26259J51720_1000060	3300003593 assembled JGI26259J51720_100006033	56481	0.48	49	Aquatic	Intertidal zone	Marine
3300003593	JGI26259J51720_1000956	3300003593 assembled JGI26259J51720_10009564	9944	0.5	35	Aquatic	Intertidal zone	Marine
3300003594	JGI26258J51719_1001151	3300003594 assembled JGI26258J51719_10011515	9243	0.5	26	Aquatic	Intertidal zone	Marine
3300003595	JGI26263J51726_1016230	3300003595 assembled JGI26263J51726_10162301	1725	0.44	13	Aquatic	Intertidal zone	Marine
3300003599	JGI26270J51728_1000102	3300003599 assembled JGI26270J51728_100010217	56481	0.48	79	Aquatic	Intertidal zone	Marine
3300003599	JGI26270J51728_1000479	3300003599 assembled JGI26270J51728_100047919	19074	0.49	57	Aquatic	Intertidal zone	Marine
3300003600	JGI26272J51733_1000094	3300003600 assembled JGI26272J51733_100009417	56481	0.48	40	Aquatic	Intertidal zone	Marine
3300003600	JGI26272J51733_1000482	3300003600 assembled JGI26272J51733_100048219	20690	0.49	151	Aquatic	Intertidal zone	Marine
3300003615	JGI26271J51732_1000447	3300003615 assembled JGI26271J51732_10004473	19331	0.48	26	Aquatic	Intertidal zone	Marine
3300003615	JGI26271J51732_1000457	3300003615 assembled JGI26271J51732_10004575	18974	0.49	27	Aquatic	Intertidal zone	Marine
3300003615	JGI26271J51732_1001110	3300003615 assembled JGI26271J51732_10011104	9874	0.5	20	Aquatic	Intertidal zone	Marine

3300003894	Ga0063241_1001009	3300003894 assembled Ga0063241_100100925	25922	0.41	1	Aquatic	Coastal	Marine
3300004109	Ga0008650_1005762	3300004109 assembled Ga0008650_10057622	4165	0.45	23	Aquatic	Intertidal zone	Marine
3300004109	Ga0008650_1086599	3300004109 assembled Ga0008650_10865992	846	0.46	15	Aquatic	Intertidal zone	Marine
3300004111	Ga0008651_10000830	3300004111 assembled Ga0008651_100008303	18436	0.48	24	Aquatic	Intertidal zone	Marine
3300004111	Ga0008651_10002812	3300004111 assembled Ga0008651_100028127	9438	0.5	17	Aquatic	Intertidal zone	Marine
3300004273	Ga0066608_1000159	3300004273 assembled Ga0066608_100015918	43499	0.48	35	Aquatic	_	Marine
3300004273	Ga0066608_1000766	3300004273 assembled Ga0066608_10007664	18514	0.49	33	Aquatic	_	Marine
3300004274	Ga0066607_1000662	3300004274 assembled Ga0066607_100066218	18442	0.48	49	Aquatic	_	Marine
3300004274	Ga0066607_1001642	3300004274 assembled Ga0066607_10016424	9963	0.5	32	Aquatic	_	Marine
3300004276	Ga0066610_10000168	3300004276 assembled Ga0066610_1000016818	46749	0.48	18	Aquatic	_	Marine
3300004276	Ga0066610_10002848	3300004276 assembled Ga0066610_100028481	9090	0.51	9	Aquatic	_	Marine
3300004277	Ga0066611_10000780	3300004277 assembled Ga0066611_1000078018	18442	0.48	23	Aquatic	_	Marine
3300004277	Ga0066611_10002212	3300004277 assembled Ga0066611_100022127	10028	0.5	13	Aquatic	_	Marine
3300004278	Ga0066609_10000109	3300004278 assembled Ga0066609_1000010917	56476	0.48	21	Aquatic	_	Marine
3300004278	Ga0066609_10000840	3300004278 assembled Ga0066609_1000084019	19214	0.49	12	Aquatic	_	Marine
3300004774	Ga0007794_10009072	3300004774 assembled Ga0007794_100090723	2985	0.41	11	Aquatic	Lentic	Freshwater
3300004806	Ga0007854_10048618	3300004806 assembled Ga0007854_100486182	2113	0.63	5	Aquatic	Lentic	Freshwater
3300004806	Ga0007854_10008257	3300004806 assembled Ga0007854_100082575	5996	0.61	8	Aquatic	Lentic	Freshwater
3300005346	Ga0074242_10791903	3300005346 assembled Ga0074242_107919032	2721	0.5	1	Aquatic	Saline	Non-marine Saline and Alkaline
3300005346	Ga0074242_11278538	3300005346 assembled Ga0074242_112785385	4555	0.49	1	Aquatic	Saline	Non-marine Saline and Alkaline
3300005408	Ga0066848_10014278	3300005408 assembled Ga0066848_100142782	2302	0.61	7	Aquatic	Oceanic	Marine
3300005422	Ga0066829_10009190	3300005422 assembled Ga0066829_100091902	3263	0.61	14	Aquatic	Oceanic	Marine
3300005551	Ga0066843_10044423	3300005551 assembled Ga0066843_100444231	1346	0.61	8	Aquatic	Oceanic	Marine
3300005592	Ga0066838_10027011	3300005592 assembled Ga0066838_100270112	1646	0.61	6	Aquatic	Oceanic	Marine
3300005596	Ga0066834_10003058	3300005596 assembled Ga0066834_100030587	7020	0.61	9	Aquatic	Oceanic	Marine
3300005838	Ga0008649_10001461	3300005838 assembled Ga0008649_1000146118	18559	0.48	13	Aquatic	Intertidal zone	Marine
3300005919	Ga0075114_10009698	3300005919 assembled Ga0075114_100096982	4293	0.39	8	Aquatic	Saline	Non-marine Saline and Alkaline
3300005939	Ga0075123_10072329	3300005939 assembled Ga0075123_100723292	1342	0.42	6	Aquatic	Saline	Non-marine Saline and Alkaline

3300006104	Ga0007882_10003798	3300006104 assembled Ga0007882_100037985	8249	0.67	11	Aquatic	Lentic	Freshwater
3300006113	Ga0007858_1021714	3300006113 assembled Ga0007858_10217141	1466	0.59	4	Aquatic	Lentic	Freshwater
3300009149	Ga0114918_10004453	3300009149 assembled Ga0114918_100044539	12161	0.45	11	Aquatic	Oceanic	Marine
3300009149	Ga0114918_10034316	3300009149 assembled Ga0114918_100343162	3579	0.46	10	Aquatic	Oceanic	Marine
3300009150	Ga0114921_10153976	3300009150 assembled Ga0114921_101539761	1578	0.48	6	Aquatic	Oceanic	Marine
3300009150	Ga0114921_10430673	3300009150 assembled Ga0114921_104306731	958	0.49	12	Aquatic	Oceanic	Marine
3300009175	Ga0073936_10159397	3300009175 assembled Ga0073936_101593972	1678	0.45	20	Aquatic	Lake	Freshwater
3300009444	Ga0114945_10068711	3300009444 assembled Ga0114945_100687111	1952	0.43	4	Aquatic	Hot (42-90C)	Thermal springs
3300009488	Ga0114925_10088983	3300009488 assembled Ga0114925_100889832	1933	0.48	9	Aquatic	Oceanic	Marine
3300009528	Ga0114920_10146211	3300009528 assembled Ga0114920_101462111	1545	0.49	4	Aquatic	Oceanic	Marine
3300009528	Ga0114920_10154608	3300009528 assembled Ga0114920_101546082	1504	0.46	6	Aquatic	Oceanic	Marine
3300009529	Ga0114919_10432047	3300009529 assembled Ga0114919_104320471	913	0.48	6	Aquatic	Oceanic	Marine
3300009529	Ga0114919_10004307	3300009529 assembled Ga0114919_100043073	11417	0.47	12	Aquatic	Oceanic	Marine
3300009529	Ga0114919_10009432	3300009529 assembled Ga0114919_100094328	7658	0.47	25	Aquatic	Oceanic	Marine
3300009529	Ga0114919_10012637	3300009529 assembled Ga0114919_100126372	6544	0.46	26	Aquatic	Oceanic	Marine
3300009548	Ga0116107_1003730	3300009548 assembled Ga0116107_10037301	7421	0.51	7	Aquatic	Wetlands	Freshwater
3300009631	Ga0116115_1048540	3300009631 assembled Ga0116115_10485401	1131	0.5	4	Aquatic	Wetlands	Freshwater
3300009691	Ga0114944_1013196	3300009691 assembled Ga0114944_10131963	2777	0.42	9	Aquatic	Hot (42-90C)	Thermal springs
3300009705	Ga0115000_10001155	3300009705 assembled Ga0115000_1000115523	23912	0.43	11	Aquatic	Oceanic	Marine
3300009788	Ga0114923_10000413	3300009788 assembled Ga0114923_1000041324	35155	0.48	16	Aquatic	Oceanic	Marine
3300009788	Ga0114923_10008664	3300009788 assembled Ga0114923_100086644	6781	0.47	16	Aquatic	Oceanic	Marine
3300009788	Ga0114923_10042446	3300009788 assembled Ga0114923_100424461	3117	0.48	13	Aquatic	Oceanic	Marine
3300009788	Ga0114923_10001294	3300009788 assembled Ga0114923_100012944	17525	0.53	11	Aquatic	Oceanic	Marine
3300009788	Ga0114923_10000757	3300009788 assembled Ga0114923_1000075722	23652	0.48	13	Aquatic	Oceanic	Marine
3300009788	Ga0114923_10212887	3300009788 assembled Ga0114923_102128872	1390	0.51	11	Aquatic	Oceanic	Marine
3300009788	Ga0114923_10251419	3300009788 assembled Ga0114923_102514191	1279	0.48	12	Aquatic	Oceanic	Marine
3300009788	Ga0114923_10384040	3300009788 assembled Ga0114923_103840401	1032	0.52	25	Aquatic	Oceanic	Marine
3300010313	Ga0116211_1016111	3300010313 assembled Ga0116211_10161113	2430	0.37	23	Aquatic	Hot (42-90C)	Thermal springs
3300010341	Ga0074045_10188987	3300010341 assembled Ga0074045_101889872	1384	0.48	5	Terrestrial	Wetlands	Soil
3300010883	Ga0133547_10012217	3300010883 assembled Ga0133547_1001221724	23288	0.43	11	_	_	_
3300011118	Ga0114922_10375316	3300011118 assembled Ga0114922_103753161	1181	0.46	12	Aquatic	Oceanic	Marine
3300011118	Ga0114922_10000660	3300011118 assembled Ga0114922_1000066037	33182	0.47	13	Aquatic	Oceanic	Marine

3300011118	Ga0114922_10009929	3300011118 assembled Ga0114922_1000992910	8668	0.38	12	Aquatic	Oceanic	Marine
3300011118	Ga0114922_10094539	3300011118 assembled Ga0114922_100945392	2563	0.51	7	Aquatic	Oceanic	Marine
3300011118	Ga0114922_10017336	3300011118 assembled Ga0114922_100173366	6504	0.46	13	Aquatic	Oceanic	Marine
3300011118	Ga0114922_10016714	3300011118 assembled Ga0114922_100167142	6621	0.47	16	Aquatic	Oceanic	Marine
3300011118	Ga0114922_10000527	3300011118 assembled Ga0114922_1000052739	36721	0.47	72	Aquatic	Oceanic	Marine
3300011118	Ga0114922_10071487	3300011118 assembled Ga0114922_100714872	2995	0.44	11	Aquatic	Oceanic	Marine
3300011118	Ga0114922_10391975	3300011118 assembled Ga0114922_103919751	1152	0.46	10	Aquatic	Oceanic	Marine
3300013089	Ga0163203_1008885	3300013089 assembled Ga0163203_10088853	2816	0.47	8	Aquatic	Lake	Freshwater
3300013089	Ga0163203_1044557	3300013089 assembled Ga0163203_10445572	1293	0.46	8	Aquatic	Lake	Freshwater
3300013098	Ga0164320_10076249	3300013098 assembled Ga0164320_100762493	1414	0.44	3	Aquatic	Hydrothermal vents	Marine
3300013101	Ga0164313_10752442	3300013101 assembled Ga0164313_107524421	801	0.37	3	Aquatic	Hydrothermal vents	Marine
3300013126	Ga0172367_10000182	3300013126 assembled Ga0172367_1000018247	96170	0.51	17	Aquatic	Lake	Freshwater
3300013131	Ga0172373_10000710	3300013131 assembled Ga0172373_1000071034	54785	0.52	13	Aquatic	Lake	Freshwater
3300013131	Ga0172373_10028983	3300013131 assembled Ga0172373_100289835	5408	0.64	31	Aquatic	Lake	Freshwater
3300013232	Ga0170573_10313230	3300013232 assembled Ga0170573_103132302	1522	0.53	1	Wastewater	Mine water	Industrial wastewater
3300014151	Ga0181539_1000813	3300014151 assembled Ga0181539_100081328	37211	0.52	11	Aquatic	Wetlands	Freshwater
3300014152	Ga0181533_1006199	3300014152 assembled Ga0181533_100619915	10985	0.53	7	Aquatic	Wetlands	Freshwater
3300014153	Ga0181527_1003838	3300014153 assembled Ga0181527_100383817	13211	0.53	5	Aquatic	Wetlands	Freshwater
3300014158	Ga0181521_10031389	3300014158 assembled Ga0181521_100313891	4041	0.51	6	Aquatic	Wetlands	Freshwater
3300014159	Ga0181530_10051393	3300014159 assembled Ga0181530_100513932	2696	0.51	5	Aquatic	Wetlands	Freshwater
3300014491	Ga0182014_10242217	3300014491 assembled Ga0182014_102422171	951	0.50	3	Terrestrial	Wetlands	Soil
3300014638	Ga0181536_10005502	3300014638 assembled Ga0181536_1000550216	12974	0.52	6	Aquatic	Wetlands	Freshwater
3300014654	Ga0181525_10015597	3300014654 assembled Ga0181525_100155974	4863	0.44	6	Aquatic	Wetlands	Freshwater
3300014654	Ga0181525_10028828	3300014654 assembled Ga0181525_100288281	3339	0.44	5	Aquatic	Wetlands	Freshwater
3300014903	Ga0164321_10149727	3300014903 assembled Ga0164321_101497271	1025	0.5	16	Aquatic	Hydrothermal vents	Marine
3300015153	Ga0179701_1030	3300015153 assembled Ga0179701_103013	16976	0.5	1	Aquatic	Creek	Freshwater
3300015370	Ga0180009_10003307	3300015370 assembled Ga0180009_1000330716	18408	0.53	11	Aquatic	Groundwater	Freshwater
3300017818	Ga0181565_10010534	3300017818 assembled Ga0181565_100105342	6974	0.35	5	Aquatic	Intertidal zone	Marine
3300017925	Ga0187856_1054136	3300017925 assembled Ga0187856_10541362	1743	0.5	4	Aquatic	Wetlands	Freshwater
3300017929	Ga0187849_1001835	3300017929 assembled Ga0187849_10018356	21642	0.51	14	Aquatic	Wetlands	Freshwater
3300017963	Ga0180437_10042574	3300017963 assembled Ga0180437_100425744	4361	0.6	6	Aquatic	Hypersaline	Non-marine Saline and Alkaline

3300017971	Ga0180438_10034967	3300017971 assembled Ga0180438_100349676	5052	0.59	8	Aquatic	Hypersaline	Non-marine Saline and Alkaline
3300017985	Ga0181576_10213006	3300017985 assembled Ga0181576_102130061	1258	0.36	5	Aquatic	Intertidal zone	Marine
3300017987	Ga0180431_10153599	3300017987 assembled Ga0180431_101535992	1811	0.59	4	Aquatic	Hypersaline	Non-marine Saline and Alkaline
3300017991	Ga0180434_10021706	3300017991 assembled Ga0180434_100217068	6288	0.48	9	Aquatic	Hypersaline	Non-marine Saline and Alkaline
3300017996	Ga0187891_1004604	3300017996 assembled Ga0187891_10046047	8877	0.51	6	Aquatic	Wetlands	Freshwater
3300018002	Ga0187868_1133556	3300018002 assembled Ga0187868_11335561	919	0.51	3	Aquatic	Wetlands	Freshwater
3300018004	Ga0187865_1051425	3300018004 assembled Ga0187865_10514252	1653	0.5	6	Aquatic	Wetlands	Freshwater
3300018016	Ga0187880_1004761	3300018016 assembled Ga0187880_100476116	9892	0.53	6	Aquatic	Wetlands	Freshwater
3300018019	Ga0187874_10002312	3300018019 assembled Ga0187874_1000231216	14810	0.52	8	Aquatic	Wetlands	Freshwater
3300018023	Ga0187889_10003899	3300018023 assembled Ga0187889_1000389911	12724	0.52	8	Aquatic	Wetlands	Freshwater
3300018024	Ga0187881_10004743	3300018024 assembled Ga0187881_100047436	10849	0.51	9	Aquatic	Wetlands	Freshwater
3300018025	Ga0187885_10055621	3300018025 assembled Ga0187885_100556213	2019	0.51	4	Aquatic	Wetlands	Freshwater
3300018026	Ga0187857_10066560	3300018026 assembled Ga0187857_100665602	1803	0.5	6	Aquatic	Wetlands	Freshwater
3300018033	Ga0187867_10016949	3300018033 assembled Ga0187867_100169492	4801	0.51	7	Aquatic	Wetlands	Freshwater
3300018080	Ga0180433_10001384	3300018080 assembled Ga0180433_1000138425	40544	0.49	14	Aquatic	Hypersaline	Non-marine Saline and Alkaline
3300018426	Ga0181566_10277647	3300018426 assembled Ga0181566_102776472	1217	0.35	6	Aquatic	Intertidal zone	Marine
3300019082	Ga0187852_1088400	3300019082 assembled Ga0187852_10884002	1379	0.5	3	Aquatic	Wetlands	Freshwater
3300020158	Ga0194038_1008083	3300020158 assembled Ga0194038_10080834	3924	0.43	50	Aquatic	Lake	Freshwater
3300020164	Ga0194037_1001670	3300020164 assembled Ga0194037_10016702	15652	0.5	33	Aquatic	Lake	Freshwater
3300020164	Ga0194037_1001667	3300020164 assembled Ga0194037_100166714	15664	0.49	29	Aquatic	Lake	Freshwater
3300020164	Ga0194037_1011260	3300020164 assembled Ga0194037_10112602	4419	0.49	26	Aquatic	Lake	Freshwater
3300020164	Ga0194037_1061635	3300020164 assembled Ga0194037_10616352	1389	0.42	11	Aquatic	Lake	Freshwater
3300021055	Ga0206226_1000122	3300021055 assembled Ga0206226_10001224	7597	0.55	7	Terrestrial	_	Deep subsurface
3300021055	Ga0206226_1000308	3300021055 assembled Ga0206226_10003086	4868	0.55	7	Terrestrial	_	Deep subsurface
3300021070	Ga0194056_10072680	3300021070 assembled Ga0194056_100726802	1264	0.48	6	Aquatic	Lake	Freshwater
3300021070	Ga0194056_10011222	3300021070 assembled Ga0194056_100112225	3876	0.41	11	Aquatic	Lake	Freshwater
3300021071	Ga0194058_10007055	3300021071 assembled Ga0194058_100070555	4741	0.41	22	Aquatic	Lake	Freshwater

3300021072	Ga0194057_10085138	3300021072 assembled Ga0194057_100851382	1162	0.42	8	Aquatic	Lake	Freshwater
3300021074	Ga0194044_10007120	3300021074 assembled Ga0194044_100071205	5165	0.41	36	Aquatic	Lake	Freshwater
3300021075	Ga0194063_10038292	3300021075 assembled Ga0194063_100382923	2343	0.47	19	Aquatic	Lake	Freshwater
3300021075	Ga0194063_10003754	3300021075 assembled Ga0194063_100037546	8973	0.42	11	Aquatic	Lake	Freshwater
3300021354	Ga0194047_10033019	3300021354 assembled Ga0194047_100330195	2446	0.48	14	Aquatic	Lake	Freshwater
3300021354	Ga0194047_10052823	3300021354 assembled Ga0194047_100528232	1840	0.47	13	Aquatic	Lake	Freshwater
3300021354	Ga0194047_10115754	3300021354 assembled Ga0194047_101157541	1124	0.49	11	Aquatic	Lake	Freshwater
3300021354	Ga0194047_10015473	3300021354 assembled Ga0194047_100154733	3821	0.47	32	Aquatic	Lake	Freshwater
3300021600	Ga0194059_1000242	3300021600 assembled Ga0194059_100024217	26862	0.53	334	Aquatic	Lake	Freshwater
3300021600	Ga0194059_1012693	3300021600 assembled Ga0194059_10126932	3053	0.48	61	Aquatic	Lake	Freshwater
3300021601	Ga0194061_1001854	3300021601 assembled Ga0194061_100185413	11930	0.56	26	Aquatic	Lake	Freshwater
3300021601	Ga0194061_1015916	3300021601 assembled Ga0194061_10159162	3006	0.48	12	Aquatic	Lake	Freshwater
3300021602	Ga0194060_10077240	3300021602 assembled Ga0194060_100772402	1870	0.49	8	Aquatic	Lake	Freshwater
3300021602	Ga0194060_10157201	3300021602 assembled Ga0194060_101572011	1200	0.47	11	Aquatic	Lake	Freshwater
3300021602	Ga0194060_10055569	3300021602 assembled Ga0194060_100555694	2287	0.5	15	Aquatic	Lake	Freshwater
3300021602	Ga0194060_10002744	3300021602 assembled Ga0194060_100027449	12523	0.5	21	Aquatic	Lake	Freshwater
3300021602	Ga0194060_10010013	3300021602 assembled Ga0194060_100100132	6232	0.51	26	Aquatic	Lake	Freshwater
3300021602	Ga0194060_10079554	3300021602 assembled Ga0194060_100795542	1838	0.66	10	Aquatic	Lake	Freshwater
3300022116	Ga0210213_1003	3300022116 assembled Ga0210213_100344	66040	0.56	1	Aquatic	Groundwater	Freshwater
3300022204	Ga0224496_10000336	3300022204 assembled Ga0224496_1000033617	33788	0.55	53	Aquatic	Sediment	Marine
3300022214	Ga0224505_10002359	3300022214 assembled Ga0224505_100023598	11110	0.54	10	Aquatic	Sediment	Marine
3300022217	Ga0224514_10083577	3300022217 assembled Ga0224514_100835771	1088	0.49	13	Aquatic	Sediment	Marine
3300022223	Ga0224501_10174201	3300022223 assembled Ga0224501_101742011	1206	0.49	7	Aquatic	Sediment	Marine
3300022223	Ga0224501_10197845	3300022223 assembled Ga0224501_101978451	1103	0.53	10	Aquatic	Sediment	Marine
3300022225	Ga0187833_10011479	3300022225 assembled Ga0187833_100114793	7512	0.62	32	Aquatic	Oceanic	Marine
3300022309	Ga0224510_10000091	3300022309 assembled Ga0224510_1000009124	76454	0.51	16	Aquatic	Sediment	Marine

Supplementary Table S4. Quality metrics for scaffolds containing the investigated *mamK* genes and ecosystem types of their metagenomic samples.

Genome name	16S rRNA scaffold ID	16S GTDB Taxonomy
Magnetococcales bacterium MAG_21055_mgc_1	-	-
Rhodospirillaceae bacterium MAG_22225_2-02_112	Ga0187833_10007879	Bacteria (domain, 51%);Proteobacteria (phylum, 50%);Alphaproteobacteria (class, 47%); unclassified_Alphaproteobacteria (order, 47%)
Rhodospirillaceae bacterium MAG_05596_2-02_51	-	-
Rhodospirillaceae bacterium MAG_05422_2-02_14	Ga0066829_1000195	Bacteria (domain, 49%);Proteobacteria (phylum, 49%);Alphaproteobacteria (class, 45%); unclassified_Alphaproteobacteria (order, 45%)
Rhodospirillaceae bacterium MAG_01419_mvb_30	-	-
Rhodospirillaceae bacterium MAG_04806_tlms_2	Ga0007854_10047206	Bacteria (domain, 69%);Proteobacteria (phylum, 66%);Alphaproteobacteria (class, 64%); unclassified_Alphaproteobacteria (order, 64%)
Rhodospirillaceae bacterium MAG_06104_tlms_034	-	-
Planctomycetes bacterium MAG_11118_pl_115	-	-
Planctomycetes bacterium MAG_17991_60	-	-
Planctomycetes bacterium MAG_18080_157	Ga0180433_1000741215	Bacteria (domain, 49%);Planctomycetota (phylum, 49%);Phycisphaerae (class, 49%); SG8-4(order,49%); SG8-4 (family, 41%);unclassified_SG8-4 (genus, 41%)
Nitrospirae bacterium MAG_10313_ntr_31	-	-
Deltaproteobacteria bacterium MAG_17929_sntb_26	-	-
Deltaproteobacteria bacterium MAG_17996_sntb_20	-	-
Deltaproteobacteria bacterium MAG_00134_naph_006	-	-
Deltaproteobacteria bacterium MAG_00241_naph_010	-	-
Deltaproteobacteria bacterium MAG_00792_naph_016	BS_KBA_SWE02_21mDRAFT_10000768	Bacteria (domain, 60%);Desulfobacterota (phylum, 58%);Desulfobacteria (class, 57%); Desulfatiglandales (order, 55%);unclassified_Desulfatiglandales (family, 55%)
Deltaproteobacteria bacterium MAG_09788_naph_37	-	-
Deltaproteobacteria bacterium MAG_15370_dsfb_81	Ga0180009_10000638	Bacteria (domain, 58%);Desulfobacterota (phylum, 57%);Desulfobacteria (class, 52%); Desulfobacterales (order, 49%);unclassified_Desulfobacterales (family, 49%)
Deltaproteobacteria bacterium MAG_22309_dsfv_022	Ga0224510_10000543	Bacteria (domain, 88%);Desulfobacterota (phylum, 88%);Desulfobulbia (class, 88%); Desulfobulbales (order, 88%); Desulfurivibrionaceae (family, 88%);UBA2262 (genus, 88%)
Deltaproteobacteria bacterium MAG_22204_dsfv_001	Ga0224496_100002163	Bacteria (domain, 53%);Desulfobacterota (phylum, 53%);Desulfobulbia (class, 52%);Desulfobulbales (order, 52%);Desulfurivibrionaceae (family, 48%);unclassified_Desulfurivibrionaceae (genus, 48%)
Pelobacteraceae bacterium_MAG_21600_9_004	-	-
Pelobacteraceae bacterium MAG_13126_9_058	-	-

Pelobacteraceae bacterium MAG_21601_9_030	Ga0194061_1000969	Bacteria (domain, 64%);Desulfuromonadota (phylum, 63%);Desulfuromonadia (class, 63%); Geobacterales (order, 63%);Pelobacteraceae (family, 58%);Pelobacter_C (genus, 52%)
Gammaproteobacteria bacterium MAG_00150_gam_010	-	-
Gammaproteobacteria bacterium MAG_00160_gam_009	-	-
Gammaproteobacteria bacterium MAG_00172_gam_018	-	-
Gammaproteobacteria bacterium MAG_00188_gam_006	-	-
Gammaproteobacteria bacterium MAG_00212_gam_1	-	-
Gammaproteobacteria bacterium MAG_00215_gam_020	-	-
Planctomycetes bacterium SCGC_JGI090-P21	A3QODRAFT_NODE-unique_58_len_8043.58	92% identity with Phycisphaerae Pla1 lineage JQ989798 in SILVA SSU Database
Omnitrophica bacterium SCGC_AG-290-C17	Ga0179701_1060	Bacteria (domain, 48%);Omnitrophota (phylum, 48%);Omnitrophia (class, 48%); Omnitrophales (order, 48%); unclassified_Omnitrophales (family, 48%)
Uncultured microorganism SbSrfc.SA12.01.D19	Ga0210213_1011	Bacteria (domain, 45%);Desulfobacterota (phylum, 42%);unclassified_Desulfobacterota (class, 42%)
Elusimicrobia bacterium NORP122	NVTF01000082.1	Bacteria (domain, 99%);Elusimicrobiota (phylum, 99%);Elusimicrobia (class, 99%); UBA1565 (order, 99%); UBA1565 (family, 99%);UBA1565 (genus, 99%)
Unclassified Nitrospina<br>Bin 25	Ga0113617_1370	Bacteria (domain, 50%);Nitrospinota (phylum, 46%);Nitrospinia (class, 46%); Nitrospinales (order, 46%);Nitrospinaceae (family, 46%);unclassified_Nitrospinaceae (genus, 46%)
Nitrospinae bacterium MAG_09705_ntspn_70	-	-
Ca. Hydrogenedentes bacterium MAG_17971_hgd_130	Ga0180438_10032718	90% identity to Candidatus Hydrogenedens YC-ZSS-LKJ63 in SILVA SSU database
Ca. Hydrogenedentes bacterium MAG_17963_hgd_111	-	-

Supplementary Table S5. 16S rRNA gene taxonomic assignments for the MAGS, SAGs, and genomes obtained in this work.
